# Supplementary figures and images for: The COMBO window: A chronic cranial implant for multiscale circuit interrogation in mice
Source: PLoS Biol. 2024 Jun 3;22(6):e3002664. doi: 10.1371/journal.pbio.3002664 (PMC11185485; doi:10.1371/journal.pbio.3002664)

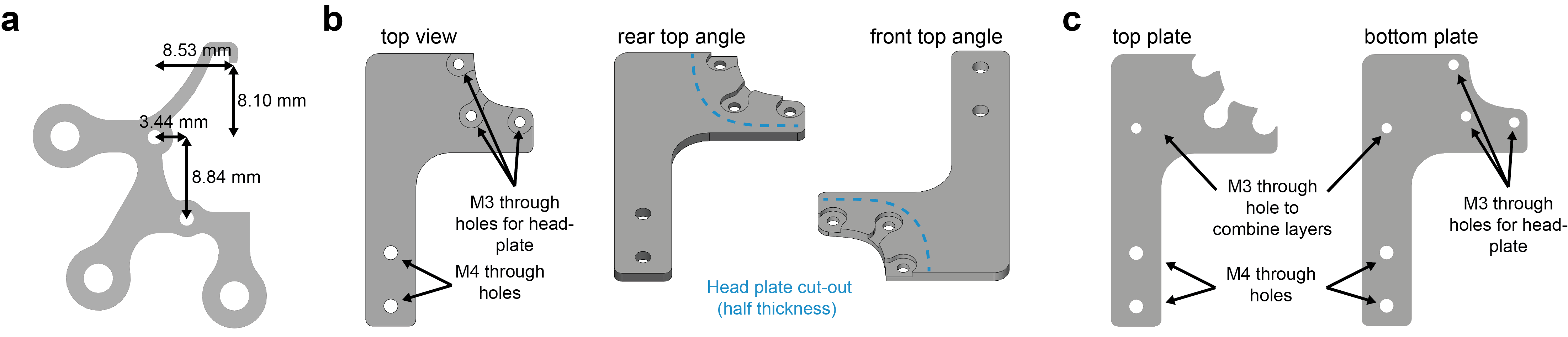

Supplement: S1 Fig — (a) Computer-aided design of the standard COMBO window head plate (S11 File). The head plate attaches to the implant via 2 M1.4 through holes at the side and rear, as well as a peg in the front. Other custom head plate designs with the same features and relative distances can also be used for head fixation. (b) The standard head plate holder design (S13 File) consists of a single metal plate with the head plate outline cut halfway through the total thickness. Threaded M3 screws are welded into the head plate holes and grinded flush with the underside of the plate. M4 through holes allow for attachment to other commercial or custom parts for further stabilization. It is recommended that a machine shop helps with the fabrication of this part. (c) An alternative head plate holder design consists of a top and bottom plate (S14–S15 Files) that can each be laser cut and joined together with no custom fabrication. M3 screws can be used to secure the 2 layers together, and M4 screws to attach the head plate holder to other commercial or custom parts. Additional M3 screws can be attached via the underside of the holder using glue/epoxy. (TIF) [file pbio.3002664.s021.tif]

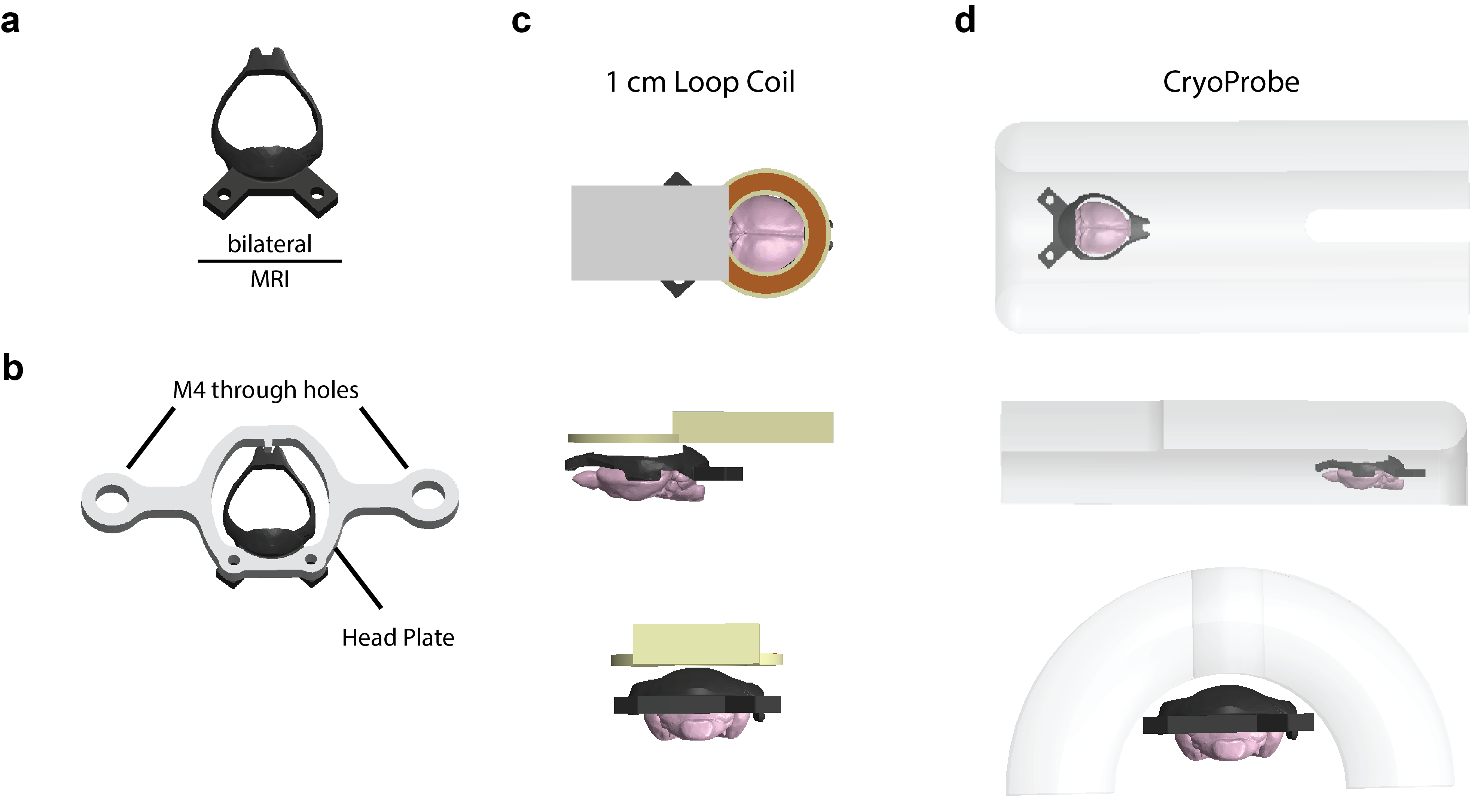

Supplement: S2 Fig — (a) Whole-brain implant version (“bilateral”) designed for magnetic resonance imaging (MRI) compatibility. Head fixation can be performed using ear bars in the scanner using this version. (b) Computer-aided design of a head plate compatible with the “bilateral” design that can be used outside of the scanner, if desired. (c, d) Schematics of the bilateral implant alongside a standard 1 cm loop coil (c) and a CryoProbe (d). As with any implant, air bubbles should be minimized when installing the implant and dental cement to avoid MR-related artifacts. (TIF) [file pbio.3002664.s022.tif]

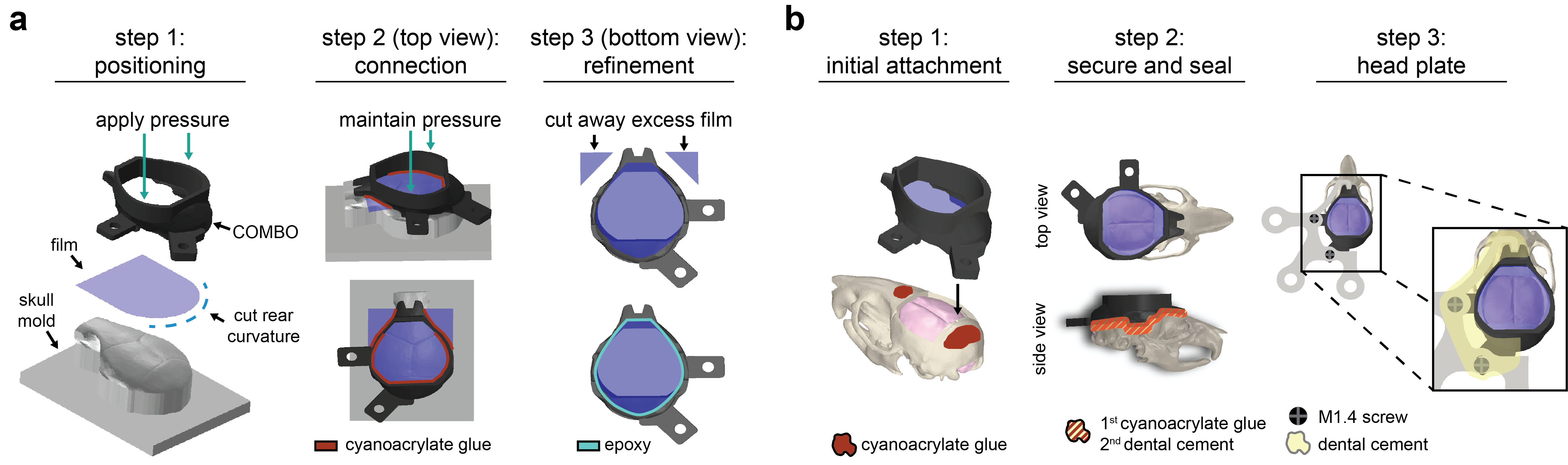

Supplement: S3 Fig — (a) Three-step diagram of the preparation of the COMBO window. Using the skull mold (S16 File) is recommended but not required for proper assembly. (b) Three-step diagram of the installation of the COMBO window after a cranial window has been created. The head plate can be installed at the same time as Steps 1 and 2 or at a later date. Detailed methods for both of the procedures are provided in S1 Appendix. (TIF) [file pbio.3002664.s023.tif]

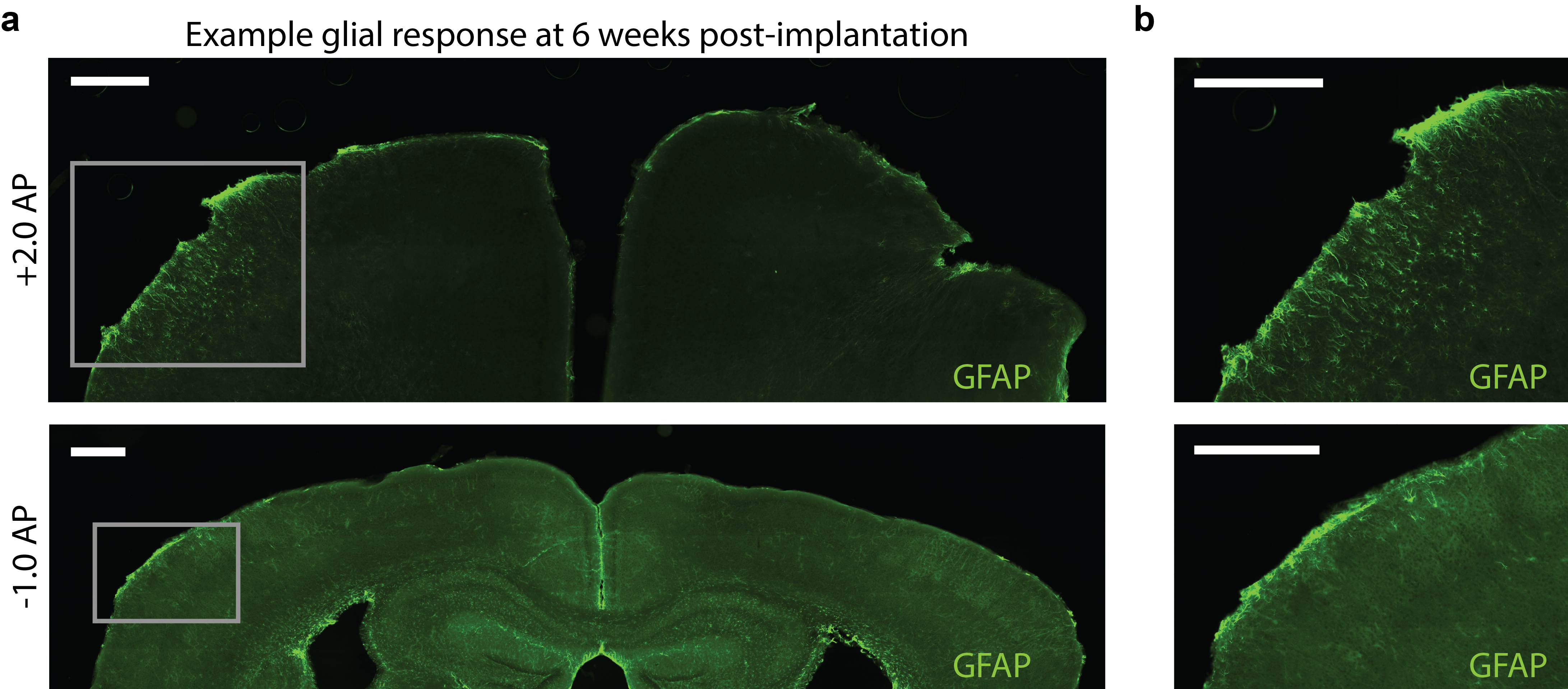

Supplement: S4 Fig — (a) Glial fibrillary acidic protein (GFAP) fluorescence in 2 example slices (top: bregma +2.0 mm AP, bottom: bregma −1.0 mm AP) of mice at 6 weeks after being implanted with the COMBO window. In both images, a localized increase of GFAP fluorescence can be seen in the left hemisphere. (b) Zoomed-in images of the elevated GFAP signal indicate that the immune response was found mostly in fibers located at or near the pial surface. Scale bars represent 500 μm. (TIF) [file pbio.3002664.s024.tif]

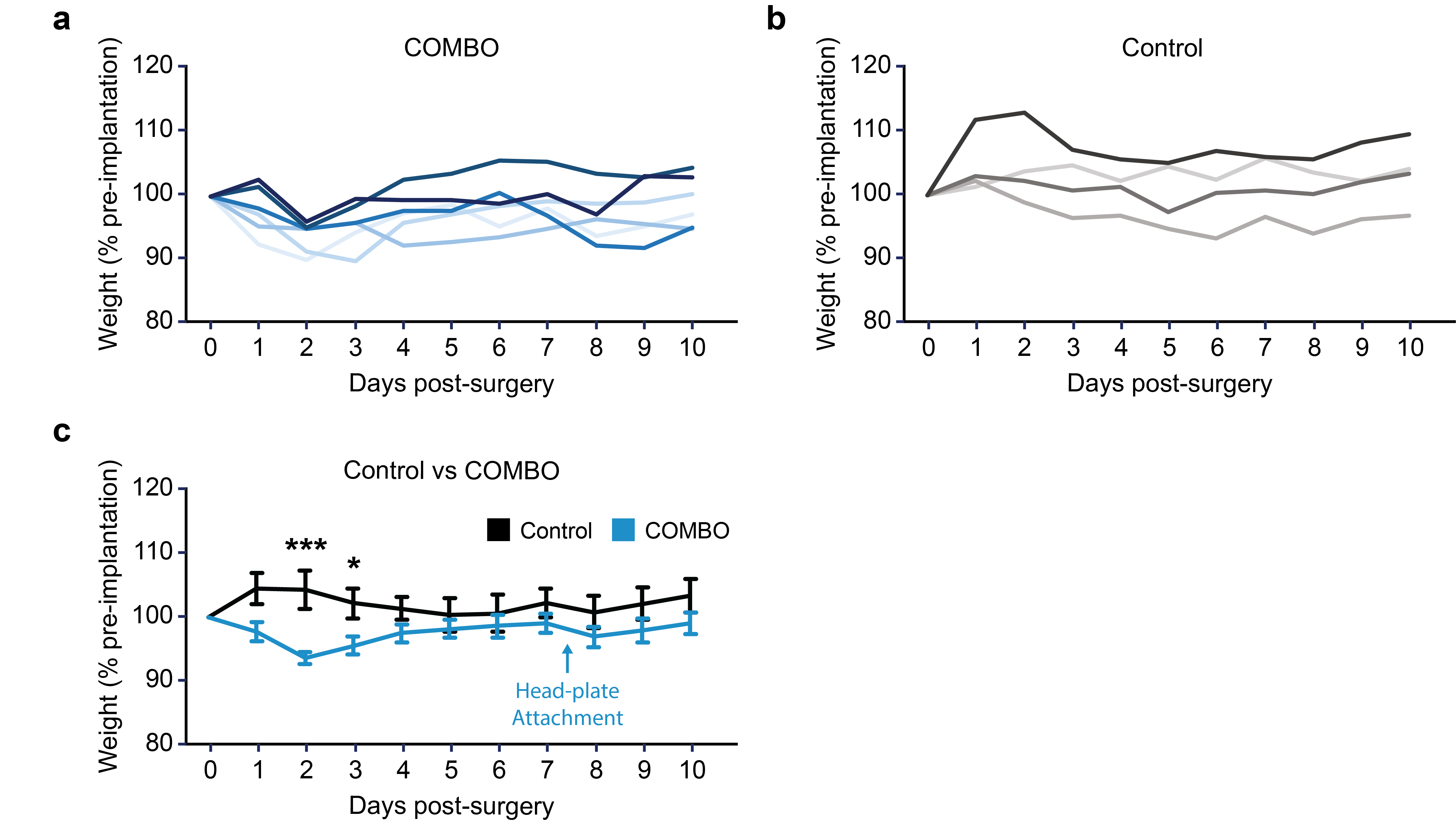

Supplement: S5 Fig — (a, b) The weight of individual animals implanted with a COMBO window (N = 6 mice) (a) and age-matched littermate controls (N = 4 mice) tracked for 10 days after installation (b). (c) Group-level weight values for animals with (blue) and without (black) a COMBO window installed. Points represent the mean ± SEM. Wilcoxon rank sum test, * p < 0.05, *** p < 0.005, uncorrected. Underlying data can be found in S6 Data and code in S6 Code. (TIF) [file pbio.3002664.s025.tif]

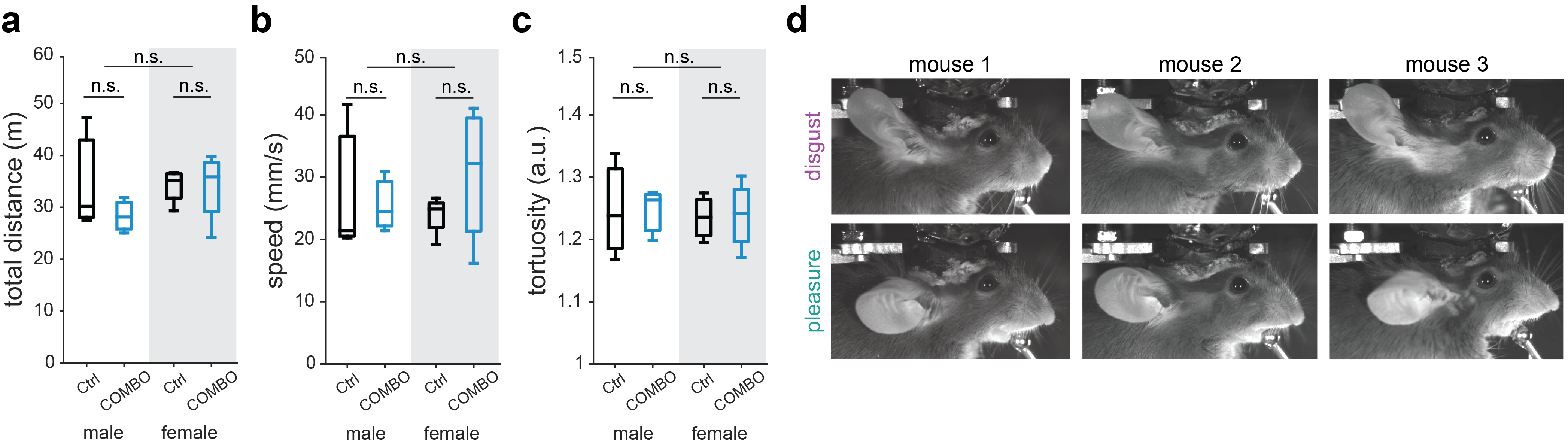

Supplement: S6 Fig — (a–c) The total distance (a), speed (b), and tortuosity (c) of control and COMBO window mice separated into males (n = 3 mice) and females (n = 4 mice). Boxplots represent the median (center line), 25th and 75th percentiles (lower and upper box), and the 1st and 99th percentile (whiskers). Two-way ANOVA on ranks with main effects of sex and cranial window. Post hoc pairwise t tests, Bonferroni corrected: n.s. p > 0.05. (d) Example prototypical disgust and pleasure facial expressions exhibited by 3 animals with a “cup” version of the COMBO window installed. Key features of the elicited disgust face include a flaring back of ear and an upturned snout, and of the elicited pleasure face include the forward movement of the ear and a downturned snout. Underlying data can be found in S7 Data and code in S7 Code. (TIF) [file pbio.3002664.s026.tif]

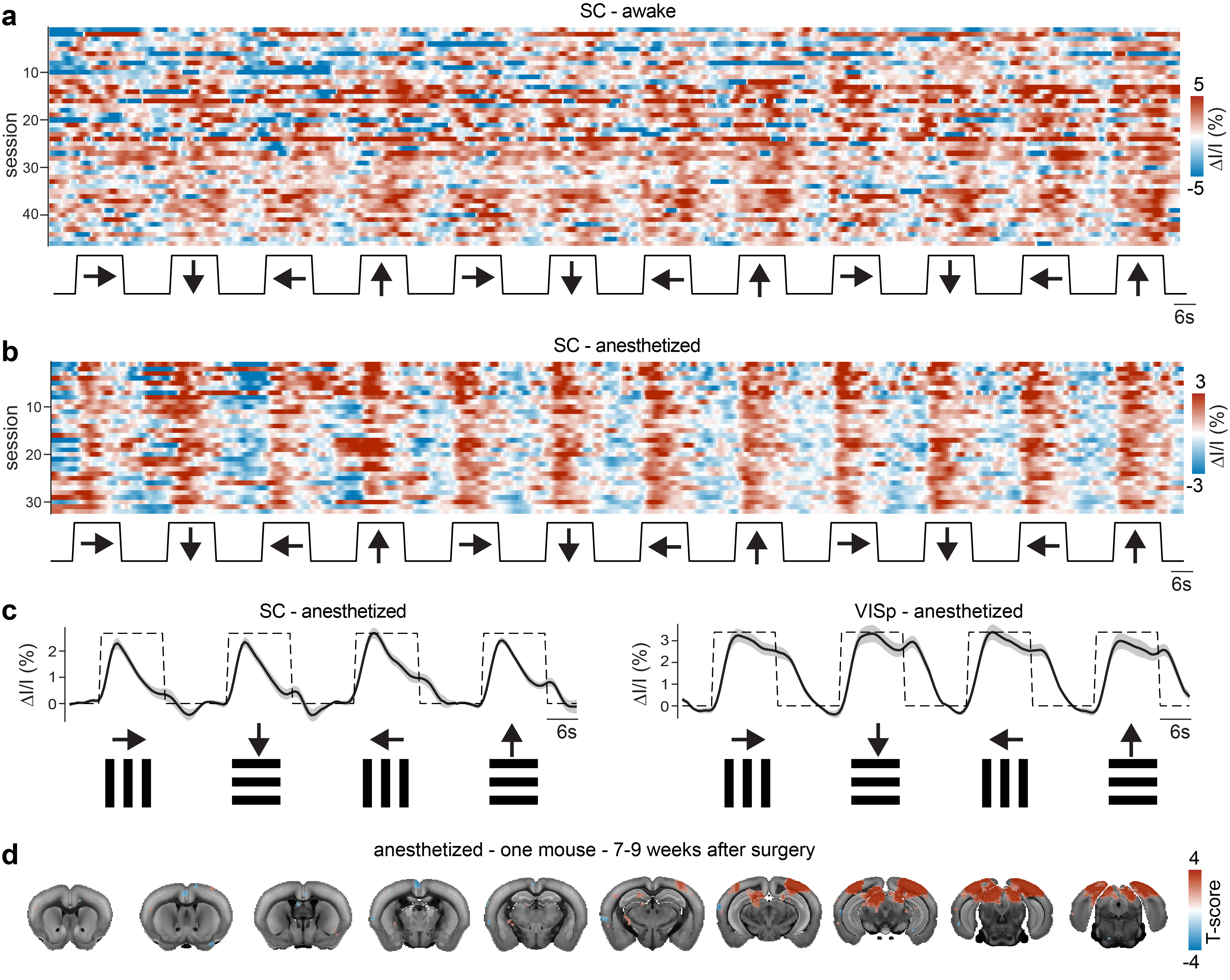

Supplement: S7 Fig — (a, b) Single-trial fUS traces from the SC plotted for each awake (a, N = 4 mice, n = 46 sessions) and anesthetized (b, N = 5 mice, n = 32 sessions) recording session. The arrows indicate the direction of the drifting grating visual stimulus. (c) fUS signal in the superior colliculi (SC) and primary visual cortex (VISp) of anesthetized mice covaries in response to drifting gratings in all 4 cardinal directions (N = 5 mice, n = 32 sessions). The dark black line and light gray shaded area represent the mean ± SEM, respectively, across sessions. (d) GLM results from 8 sessions of a single mouse recorded 7–9 weeks after being implanted with a COMBO window overlaid on the Allen Brain Atlas. Only voxels with an average T-score > 2 are displayed. Underlying data can be found in S8 Data and code in S8 and S11 Codes. (TIF) [file pbio.3002664.s027.tif]

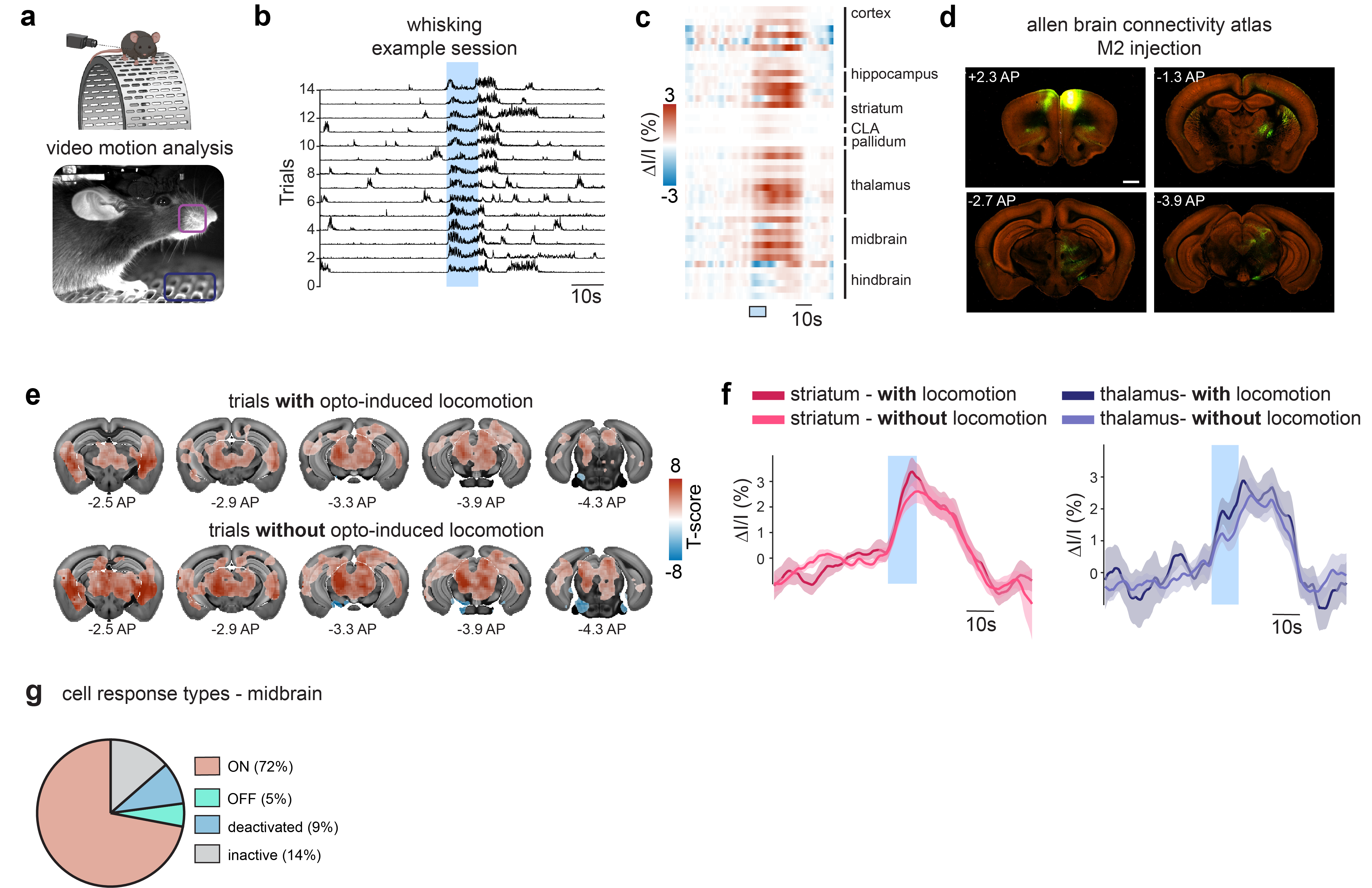

Supplement: S8 Fig — (a) Facial videography was used to monitor animal behavior on a running wheel. Regions-of-interest (ROIs) placed over the whisker pad (violet) and wheel (green) were utilized to capture whisking activity and locomotion, respectively, induced by optogenetic stimulation of the secondary motor cortex (M2). (b) Consecutive trials from the same example session as in Fig 5C showing a robust and reliable increase in whisking in response to optogenetic activation of M2. Each trial was z-scored to a pre-stimulus baseline and rescaled between 0 and 1 for visualization purposes. (c) Region-wise segmented results of the optogenetically induced fUS activity. Regions are sorted by brain area, and only significantly modulated (correlation between stimulus timing and fUS signal) regions are included (significantly different from zero across sessions, p < 0.01, FDR-corrected). (d) Example coronal slices from the Allen Brain Connectivity Atlas (connectivity.brain-map.org/projection/experiment/287995889). AAV tracings after injection into the M2 (1) show widespread axonal projections from M2 to the striatum, the thalamus, and the midbrain (AP +2.30 mm to AP −3.90 mm). Scale bar represents 1 mm. (e) GLM analysis of fUS data in response to optogenetic stimulation of M2 (N = 3 mice, n = 15 sessions). In contrast to Fig 5E, here the trials were separated according to a strong or weak locomotor response (see Methods for threshold definition) to optogenetic stimulation. Only the voxels with T-scores significantly different from zero across sessions (p < 0.05, FDR-corrected) are shown. (f) Group-level fUS traces from the striatum (left) and thalamus (right) for trials with and without animal locomotion. The dark lines and lighter shaded areas represent the mean ± SEM, respectively, across sessions. (g) Pie chart showing the proportion of different cell response types observed in the midbrain (see Methods for cell response type definition). Underlying data can be found in S9 Data and code in [file pbio.3002664.s028.tif]

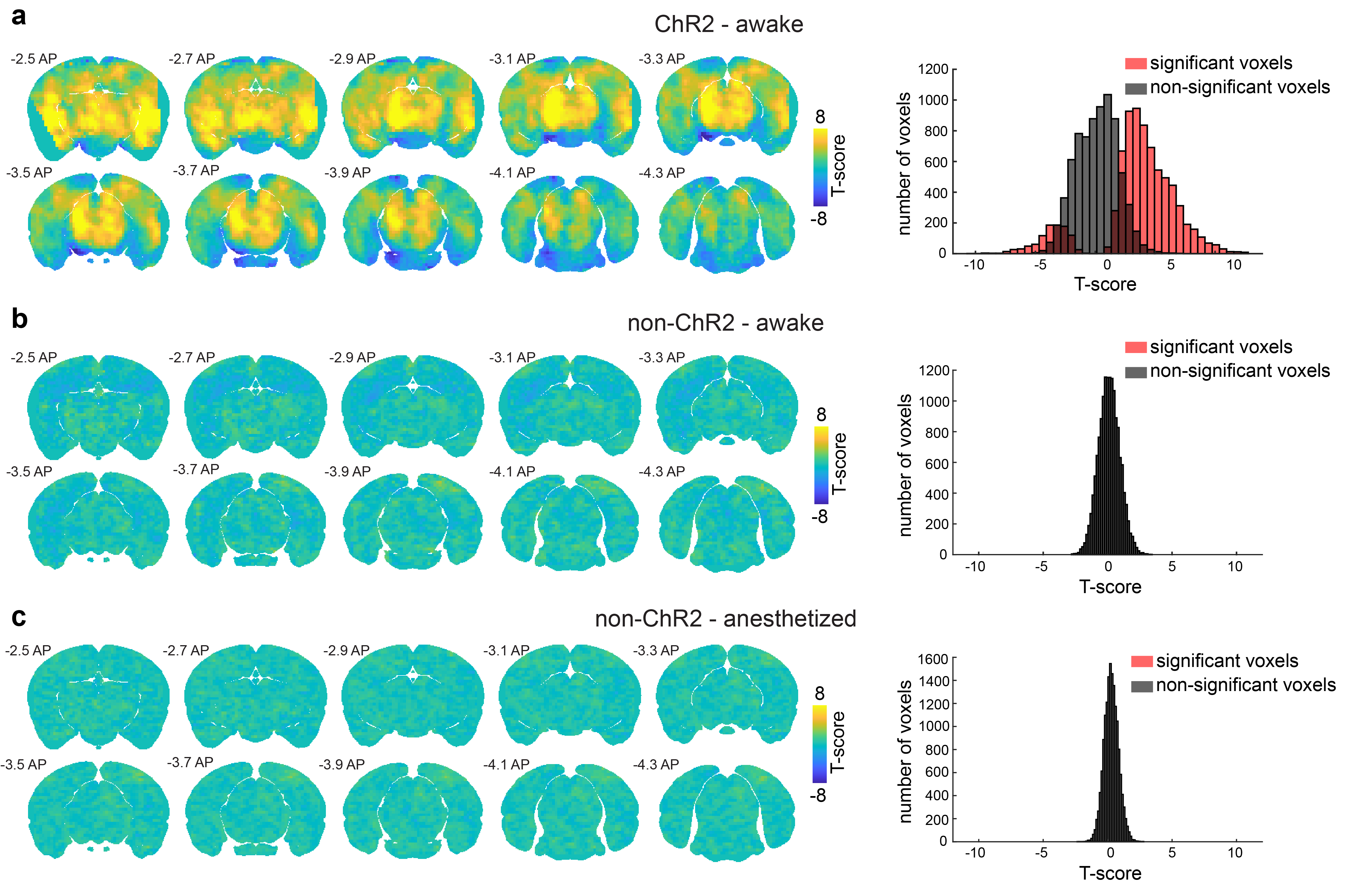

Supplement: S9 Fig — (a) Optogenetic activation of M2 results in widespread activation in ChR2-expressing mice. Average T-scores at indicated positions (distance to Bregma) are shown without thresholding (left, N = 3 mice, n = 16 sessions). Histograms of the same T-scores color-coded by significance (p < 0.05, FDR-corrected) (right). (b, c) Same as (a) for animals injected with an AAV9-CamkIIa-EGFP control virus in M2. Low T-scores and no significant voxels (p < 0.05, FDR-corrected) are observed for both the awake (N = 3 mice, n = 12 sessions) and anesthetized (N = 3 mice, n = 14 sessions) conditions. Underlying data can be found in S10 Data and code in S10 Code. (TIF) [file pbio.3002664.s029.tif]
