# Supplementary material for: The COMBO window: A chronic cranial implant for multiscale circuit interrogation in mice
Source: PLoS Biol. 2024 Jun 3;22(6):e3002664. doi: 10.1371/journal.pbio.3002664 (PMC11185485; doi:10.1371/journal.pbio.3002664)
Supplement: S1 Table — (DOCX) [file pbio.3002664.s030.docx]

**S1 Table.** Open field foraging task statistical analysis

| **Parameter** | **Statistical Test** | **Comparison** | **Post Hoc Test** | **P-value** |
| --- | --- | --- | --- | --- |
| Total Distance | Two-way ANOVA on ranks  COMBO vs Ctrl  F(1,13) = 0.59, p = 0.46  Male vs Female  F(1,13) = 1.26, p = 0.29 | COMBO vs Ctrl  (Male only) | Bonferroni | p = 1.00 |
|  |  | COMBO vs Ctrl  (Female only) | Bonferroni | p = 1.00 |
|  |  |  |  |  |
| Speed | Two-way ANOVA on ranks  COMBO vs Ctrl  F(1,13) = 0.16, p = 0.70  Male vs Female  F(1,13) = 0.05, p = 0.82 | COMBO vs Ctrl  (Male only) | Bonferroni | p = 1.00 |
|  |  | COMBO vs Ctrl  (Female only) | Bonferroni | p = 1.00 |
|  |  |  |  |  |
| Tortuosity | Two-way ANOVA on ranks  COMBO vs Ctrl  F(1,13) = 0.17, p = 0.69  Male vs Female  F(1,13) = 0.12, p = 0.74 | COMBO vs Ctrl  (Male only) | Bonferroni | p = 1.00 |
|  |  | COMBO vs Ctrl  (Female only) | Bonferroni | p = 1.00 |
