# Supplementary material for: The COMBO window: A chronic cranial implant for multiscale circuit interrogation in mice
Source: PLoS Biol. 2024 Jun 3;22(6):e3002664. doi: 10.1371/journal.pbio.3002664 (PMC11185485; doi:10.1371/journal.pbio.3002664)
Supplement: S2 Table — (DOCX) [file pbio.3002664.s031.docx]

**S2 Table.** Facial expression statistical analysis

| **Parameter** | **Statistical Test** | **Comparison** | **P-value** |
| --- | --- | --- | --- |
| Disgust | Wilcoxon rank sum test | Quinine vs Neutral | p = 0.0001 |
|  |  | Sucrose vs Neutral | p = 1.00 |
|  |  |  |  |
| Pleasure | Wilcoxon rank sum test | Quinine vs Neutral | p = 0.92 |
|  |  | Sucrose vs Neutral | p = 0.0011 |
