# Supplementary material for: The COMBO window: A chronic cranial implant for multiscale circuit interrogation in mice
Source: PLoS Biol. 2024 Jun 3;22(6):e3002664. doi: 10.1371/journal.pbio.3002664 (PMC11185485; doi:10.1371/journal.pbio.3002664)
Supplement: S3 Table — (DOCX) [file pbio.3002664.s032.docx]

**S3 Table.** Author Contributions

|  | **BJE** | **DS** | **PW** | **BJ** | **BS** | **AR** | **NG** | **TF** | **EM** |
| --- | --- | --- | --- | --- | --- | --- | --- | --- | --- |
| **Conceptualization** | **X** | **X** |  |  |  |  |  |  | **X** |
| **Data Curation** | **X** | **X** | **X** |  |  |  |  |  |  |
| **Formal Analysis** | **X** | **X** |  |  |  |  |  |  |  |
| **Funding Acquisition** | **X** | **X** |  |  |  |  | **X** | **X** | **X** |
| **Investigation** | **X** | **X** | **X** | **X** | **X** | **X** |  |  |  |
| **Methodology** | **X** | **X** | **X** | **X** |  |  | **X** | **X** | **X** |
| **Project Administration** | **X** |  |  |  |  |  |  |  | **X** |
| **Resources** |  |  |  |  |  |  | **X** | **X** | **X** |
| **Software** | **X** | **X** |  |  |  |  |  |  | **X** |
| **Supervision** | **X** |  |  |  |  |  | **X** | **X** | **X** |
| **Validation** | **X** | **X** | **X** |  |  |  |  |  | **X** |
| **Visualization** | **X** | **X** |  |  |  |  |  |  | **X** |
| **Writing - Original Draft** | **X** | **X** |  |  |  |  |  |  | **X** |
| **Writing - Review & Editing** | **X** | **X** | **X** |  |  |  |  | **X** | **X** |
