# Supplementary material for: The COMBO window: A chronic cranial implant for multiscale circuit interrogation in mice
Source: PLoS Biol. 2024 Jun 3;22(6):e3002664. doi: 10.1371/journal.pbio.3002664 (PMC11185485; doi:10.1371/journal.pbio.3002664)
Supplement: S1 Appendix — (DOCX) [file pbio.3002664.s033.docx]

**S1 Appendix: COMBO window preparation and installation protocol**

**1. COMBO Window Preparation**

1.1. Download and print one of the COMBO window files (**S1-10 Files**) and the Brain_mold.stl (**S16 File)** using a 3D printer.

NOTE: If the 3D printer utilizes supports when printing, they should be placed on the top of the implant frame and on the bottom of the skull mold to ensure smooth contact surfaces between these two parts. The supports should be cut away and the contact points sanded smooth.

1.2. Thread the two holes of the implant frame using a M1.4 tap. Due to animal safety and physical difficulty, these holes should not be threaded after implantation.

1.3. Cut a square of film (0.125 mm thickness) slightly bigger than the implant frame. Cut a round edge on one side of the film to fit the curvature at the posterior end of the implant frame (**S3a Fig, step 1**).

1.4. Place the film on the bottom mold (round part at the back) with the implant frame on top. Apply pressure at the edges of the implant frame downward onto the mold. Try to minimize buckling and ensure that there are no gaps between the film and the implant frame (**S2a Fig, step 1**). Multiple attempts to achieve proper positioning may be needed.

1.5. While maintaining pressure, apply a conservative amount of tissue adhesive (low-viscosity cyanoacrylate glue) to the interior rim of the implant frame to attach the film. It is also recommended to apply super glue (high-viscosity cyanoacrylate glue) to the exterior of the frame where any excess film protrudes (**S3a Fig, step 2**). After a few minutes (to allow the glue to dry), if these steps have been performed correctly, the implant frame and the film can be removed from the mold together. Other types of glue can also be placed on various interior/exterior edges of the frame according to what is easiest for the user.

NOTE: Avoid covering the film with glue as this can affect the transparency of the window.

1.6. Apply a layer of epoxy to the border of the interface between the film and the implant frame (**S3a Fig, step 3**).

1.7. Let the epoxy dry overnight and remove the excess film using a scalpel on the following day. Then, file the hardened epoxy as close to the implant frame as possible (**S3a Fig, step 3**). Repeat steps 1.6 – 1.7 until the film is fully secured to the implant frame with no gaps, and is flush with the surface.

NOTE: Before implant installation, ensure that the implant is thoroughly cleaned using 100% ethanol.

1.8. Download the Head_plate.dwg file (**S11 or S12 File**) and laser cut this shape from 1.5 mm stainless steel. A brushed finish will increase the grip of the dental cement.

**2. COMBO Window Installation**

**2.1. Cranial Window Surgery**

2.1.1 Prepare the animal for surgery according to locally approved animal licenses and secure the animal’s head using a bite bar or stereotaxic frame.

2.1.2. Using sterile scissors cut a 2 cm midline incision through the scalp and periosteum to expose the skull. This incision should reach from just behind the ears to the middle of the eyes.

2.1.3. Detach the periosteum using a cotton-tipped applicator. Then, detach the temporalis and trapezius muscles on the sides and rear of the skull, respectively, using scissors or forceps. Try to maximize the surface area of exposed skull to allow firm attachment of the implant in later steps. Ensure that the skull surface is dry using cotton tipped applicators (use 0.3% H2O2 if necessary).

NOTE: Avoid damaging vessels behind the eyes and at the rear of the skull when detaching the muscles. Damaging such vessels can cause large bleeds and will reduce the animal’s chance of survival.

2.1.4. Push the muscles down and secure them to the skull in place using tissue adhesive. Make sure that no gaps remain and that, as mentioned above, as much of the skull stays exposed as possible. Clean debris from the skull surface with a wet cotton-tipped applicator and dry with compressed air.

2.1.5. Set hand drill to ~5000 rotations per minute and mark an outline of the craniotomy on the skull by drilling superficially. Briefly, the extent of the craniotomy can extend the full width of the skull and from the rostral rhinal vein (anterior) to the transverse sinus (posterior) (or even further to the end of the cerebellum). Additional details regarding steps 2.1.5. – 2.1.7. can be found in [1,2].

2.1.6. Continue to deepen the outline by repeatedly moving the drill over the initial groove until the bone island is “floating” on top of the brain. Use compressed air to gently blow away bone debris as needed. Occasionally apply cool saline to the skull to prevent overheating.

2.1.7. To remove the skull, use forceps (e.g. 90 degree) to lift the anterior edge of the bone towards the posterior end. For this, cover the skull with buffer or saline and gently lift the bone island little by little until the soft tissue connections have fully detached from the dura and vessels. The dura should be left intact.

NOTE: There are strong soft tissue connections between the skull and dura along the superior sagittal sinus. Removing the skull too quickly and under too dry of conditions can rip the SSS and cause a potentially fatal bleed. This step can take up to 15 minutes or more for correct detachment without major bleeding.

2.1.8. After removal of the bone, clean the edges of the craniotomy and ensure that the residual bone surface is dry before proceeding with the next steps. Keep the dura moist at all times.

**2.2 Implant Attachment and Sealing**

2.2.1. Rinse the ethanol-cleaned implant with saline and let it dry on a paper towel.

2.2.2. Place a small amount of super glue on the skull, anterior and posterior to the cranial window. Gently place the implant on top of the cranial window, ensuring that there is secure contact with the areas covered with super glue (**S3b Fig, step 1**). The implant should hug the sides and the rear of the skull in this step.

NOTE: Some of the film may make direct contact with the dura during this step, but full contact is not necessary at this point. If sealed properly, the window will fill up with cerebrospinal fluid within a few days (**Fig 1b**).

2.2.3. Once the implant feels secure, place additional super glue in the gaps between the implant and the skull to fully secure and seal the implant to the skull (**S3b Fig, step 2**).

NOTE: There is generally less skull exposed at the anterior parts of the head, and it can therefore be difficult to properly seal this section (especially around the eyes). If these sections are not properly sealed, dental cement can easily fall through this gap and onto the brain surface in step 2.2.4.

2.2.4. Prepare dental cement according to the manufacturer’s instructions and apply generously around the circumference of the implant and skull (on top of the glue) (**S3b Fig, step 2**). Ensure that there are no gaps between the implant and skull. Contact with the sutured skin will largely prevent the animal from opening wounds.

2.2.5. Attach the head-plate to the implant with two M1.4 screws (**S3b Fig, step 3**). Prepare additional dental cement and apply to the two screws and front peg to ensure that the head-plate is locked in place on the implant. Additional cement can be placed in the gap between the head-plate and implant for a firmer connection (**S3b Fig, step 3**). This step can also be performed in a second surgery after recovery from the craniotomy and implant installation.

2.2.6. Apply silicone (e.g. Kwik-Cast silicone sealant) on top of the film to protect it until imaging.

2.2.7. Reverse the anesthesia and provide post-operative care according to locally approved animal licenses for the required number of days. When the animal is returned to its home cage, it is strongly recommended to remove any overhead gratings in the home cage to avoid interference with the implant/head-plate, and place additional feed on the floor of the cage.

**3. Head Fixation and Imaging**

3.1. We propose two assembly methods of the head fixation system for securing the mouse to the experimental setup. For the first (preferred) design, download the Head_plate_holder.sldprt file (**S13 File**) and manufacture the shape from 2 mm stainless steel from an external provider or workshop. Alternatively, the Head_plate_holder_top.dwg and Head_plate_holder_bottom.dwg files can be used together to construct the same design (**S14-15 Files**). This approach has the advantage of being compatible with laser cutting. Each part should be laser cut from 1 mm stainless steel and secured together via the rear mounting holes (M4 through holes). In either case, thread the fixation holes (M3 through holes) with an M3 tap and feed M3 screws (with low profile heads) upward through each. Alternatively, the M3 screws can be permanently secured with epoxy or M3 studs can be welded in place. The head fixation should be secured in the behavioral setup prior to the start of habituation. If using the “bilateral” implant version, head fixation can be performed either with ear bars (during anesthetized recordings) or using the bilateral head plate (**S12 File**), which is compatible with standard M4 screw connections.

3.2. Animals should be handled for three days for 10 - 15 min per day prior to being trained on a particular behavioral setup. On the first day of habituation to the setup, allow the mouse to first explore as performed previously. After 5 - 10 minutes, position the animal into alignment with the head fixation screws. Gently lift the head-plate with a pair of forceps onto the pins while supporting the body of the animal with the other hand. Tighten M3 nuts on top of the pins to secure the head-plate, first by hand and then using a socket driver. The duration of a habituation session should increase each day for at least five days.

3.3. On days in which imaging takes place, remove the silicone window protection, and wash the surface of the film with saline and dry with a cotton-tipped applicator. After imaging is complete, it is recommended that the silicone window protection be replaced (while the animal is still head-fixed) until the next imaging session.

**4. References**

1. Brunner C, Grillet M, Urban A, Roska B, Montaldo G, Macé E. Whole-brain functional ultrasound imaging in awake head-fixed mice. Nature Protocols. 2021;16: 3547–3571. doi:10.1038/s41596-021-00548-8

2. Hattori R, Komiyama T. Longitudinal two-photon calcium imaging with ultra-large cranial window for head-fixed mice. STAR Protocols. 2022;3. doi:10.1016/J.XPRO.2022.101343
