# Supplementary material for: The COMBO window: A chronic cranial implant for multiscale circuit interrogation in mice
Source: PLoS Biol. 2024 Jun 3;22(6):e3002664. doi: 10.1371/journal.pbio.3002664 (PMC11185485; doi:10.1371/journal.pbio.3002664)
Supplement: Attachment — Submitted filename: COMBO window paper review.pdf [file pbio.3002664.s055.pdf]

This paper introduces the COMBO window - an open-source method to for creating 3D printed customized cranial windows that all calcium imaging and fUS. This work builds on previous work done for wide field cortical imaging that uses a similar approach to those developed previously. The relative advance here is that the authors provide several different versions of the polymer cranial windows which could be useful in a number of different contexts. There is also an increased coverage in terms of the FOV. The authors also show fUS imaging and calcium imaging. Overall, this is a work that could be useful to a number of labs.

I do have some comments and concerns that should be addressed/ modified before publication.

1. A central argument for the development of the COMBO window is that it is uniquely developed for fUS imaging. The authors argue that the previous cranial windows with transparent films (such as the paper cited i.e., Ghanbari et al and other derivatives) are incompatible with fUS imaging (or as stated in the paper 'have issues'). It is unclear whether the authors tested such implants for fUS imaging, or if there is literature that suggest incompatibility. The only difference between the two designs is the use of the polymer films - Ghanbari et al used PET films, whereas this work uses PMP films.

Is there something specific about PMP that helps with fUS imaging?

Did the authors evaluate the use of PET for fUS imaging? If so what were the negative results, and how did they compare with PMP films? If there is no such comparative experiment conducted, it is unclear how the authors conclude previous window implants using PET films have an issue.

If there no evidence of issue either from previous studies or any experiments that the authors conducted, then this central argument of this paper needs to be watered down - it is fine to simply state this approach is compatible with fUS and that other approaches may work as well, it just not been tested.

The authors show For a paper that is touting a new kind of cranial window technology, there is very little characterization of the long term effect of the window implantation on the brain. This information will provide potential users context on feasibility of implementation. Two aspects that need to be described more:

The authors state that the COMBO windows provide increased imaging coverage. While the analysis of orofacial behavior is nice, what is more critical is to provide data on success rate of surgical implantation. This will provide potential users important context on the feasibility of the COMBO window approach. Several questions arise:

1. How may mice were implanted? What is the overall success rate of these surgeries?
2. What are typical failure points?
3. How long do these windows last? What are the neuroimmune responses that occur over time?
4. Cranial window implants are often accompanied by dura thickening, bone regrowth and general occlusion of the FOV. Did the authors encounter these issues. If so these outcomes with n values need to be reported.

Unclear why the authors do the analyses of free behavior - as the imaging is anyway done in headfixed animals…

With the increased coverage, what is a the effect of motion artifacts if any on imaging/ephys. The authors correct for lateral displacement in x and y, but there is no comment on the z displacement.

Some of the experiments are under powered - n=2 for optogenetic experiments, n =2 electrophysiology experiments. How well does the approach work?

For calcium imaging, the author developed a flat version. How large of a coverslip was used in this case? One of the issues is that implanting something like this over a very large area will results in several tens of mm^3 of brain tissue being displaced. While the experiments were done on 3 animals, there is no data provided on histology post implantation.
