# Supplementary material for: The COMBO window: A chronic cranial implant for multiscale circuit interrogation in mice
Source: PLoS Biol. 2024 Jun 3;22(6):e3002664. doi: 10.1371/journal.pbio.3002664 (PMC11185485; doi:10.1371/journal.pbio.3002664)
Supplement: Attachment — Submitted filename: EdelmanSiegenthaler2023_ResponseToReviewers_R1_Final.docx [file pbio.3002664.s056.docx]

PBIOLOGY-D-23-02796R1 Author Response

We thank the reviewers for their thoughtful comments. We have carefully considered all of the comments and have addressed all concerns, and believe the manuscript is now substantially improved. In particular, we performed new experiments and analysis for the opto-fUS section of the paper. As suggested by the reviewers, we added to our opto-fUS dataset three non-ChR2 control animals (N = 3 mice, n = 12 sessions anesthetized, n = 14 sessions awake) in addition to one ChR2 animal (N = 1, n = 4 sessions awake) to bring the total to three (N = 3 mice, n = 16 sessions). We have also created an additional version of the COMBO window that is physically compatible with MRI. Finally, we now provide more details on the surgical success rate and long-term impact of the COMBO window, including a 10-day comparison of animal weight for those with and without a COMBO window installed. Please find below the updated manuscript text in red and our responses to the comments in blue. All relevant text/figures have also been integrated into the clean and marked up versions of the associated manuscript.

**Reviewer 1**

The manuscript by Edelman and colleagues (PBIOLOGY-D-23-02796R1) presents the "COMBO" window - a versatile solution for a chronic cranial window, which enables multimodal investigation of the mouse brain. The COMBO window is a designed for enabling access to most of the brain, with durability for long-term chronic investigations, and with flexibility to accommodate many different recording/manipulation modalities such as functional ultrasound (fUS), two-photon/optical imaging, electrophysiological recordings, and optogenetic manipulations.

The authors first tested the durability and compatibility of their COMBO setup in terms of long-term inflammation, freely-moving behavior, and preservation of facial expressions. They then proceed to show proof-of-principle of the COMBO window's versatility by probing awake/anesthetized visual activity via fUS, 2P imaging of specific neurons during locomotion, optogenetic manipulation of pretargeted brain areas, and even electrophysiological recordings. In all cases, the authors were successful in clearly demonstrating both the durability/applicability of the setup.

Overall, I find that this work is a tour-de-force of what's possible in terms of multimodal investigation of brain activity, and the COMBO window presented here represents the state-of-the-art in durable chronic implants for the mouse. I have no doubt that the freely available designs, including drawings and instructions, will represent a significant resource for the community and increase the reproducibility and quality of studies employing chronic implants. In addition, having such a resource can encourage others to engage/enter the field. The paper is also written exceptionally well and is easy to follow.

**Response:** We thank the reviewer for the positive comments and support for this work. We also hope that the open-source files and instructions can impact the community and help the COMBO window be adopted by new users.

**R1.1.** One thing I haven't seen in this work is a more direct comparison against any other available implants (except perhaps for coverage issues): could the authors discuss advantages / disadvantages compared with other types of implants?

**Response:** We thank the reviewer for this comment. This point is difficult to address experimentally, given the large number of designs available. Furthermore, any test that we perform would likely be favorable to the COMBO window simply due to our extensive experience and expertise with this design. As mentioned in the ***Introduction*** section, popular glass implants such as the crystal skull are not compatible with fUS due to the strong attenuation of ultrasound waves. When compared to previously published plastic implants, we aimed to increase the brain coverage with the COMBO window, which is easy to quantify and is discussed. There are two other advantages that we pushed for with the COMBO window: (1) improved access to behavioral readouts and, (2) a practical solution to combine with optogenetics. In particular, these aspects have neither been quantified nor discussed in depth for other plastic implants, such as the See Shell, making a quantitative comparison difficult. Similarly, the disadvantages of the COMBO window compared to other implants are difficult to quantify, however, we believe that the surgical complexity and duration is increased due to the additional brain coverage and expanded size of the cranial window.

Following the reviewer’s advice, we highlight these points in a new paragraph in the ***Discussion*** section, as follows:

“To achieve compatibility of the implant with multiple techniques, we chose a polymer-based design for the COMBO window. Compared to glass, plastic does not attenuate ultrasound and allows small perforations to be made for electrophysiology. Additionally, the flexibility of plastic enables a tight fit to the curvature of the exposed mouse brain, and promotes a low impact on the brain and long-lasting window transparency. The COMBO window provides a ~ 90mm^2^ opening, which is a notable increase in field of view over alternative implant designs that provide access to 45 – 75 mm^2^ of the dorsal cortex (T. H. Kim et al. 2016; Ghanbari et al. 2019), as well as an improvement in reproducibility compared to other preparations that require window customization for each animal (Boido et al. 2019; Tournissac et al. 2022; Hattori and Komiyama 2022). With this being said, the larger cranial window associated with the COMBO window comes with the cost of potentially reduced stability (see below) and a more difficult surgical procedure. Despite the large size of the cranial window and in contrast to many other preparations (É. Macé et al. 2018; Boido et al. 2019; Edelman et al. 2021; Tournissac et al. 2022; Hattori and Komiyama 2022), we ensured that the COMBO window and custom-made head-fixation allow unobstructed readouts of mouse behavior, which constitutes a growing need in system neuroscience studies. Fine analysis of facial movements, for example, are widely used to characterize arousal or emotion states in head-fixed animals (Dolensek et al. 2020; Moëne and Larsson 2023). Additionally, compared to other previously published implant designs, we provide off-the-shelf solutions for many combinations of neural interrogation tools that make custom adjustments by the users, i.e. due to different regions of interest, unnecessary.”

**R1.2.** While I highly appreciate that the authors tested preservation of behaviours and local inflammatory responses, would it make sense to look at more global metrics? E.g., corticosteroids in the blood, overall weight loss, reproduction, etc.?

**Response:** Thank you for raising this important point. We agree that demonstrating the absence of an inflammatory response at the site of surgery does not exclude an elevated global inflammation or increased stress. In our original submission we included results from an open field task, which we used to quantify various aspects of locomotion. However, the open field is also a traditional assay used to infer stress levels in mice. In general, mice spend more time in the center when they exhibit low levels of anxiety/stress, and more time near the walls with high levels of anxiety/stress. We can assess the latter using a measure called “thigmotaxis” which is defined as 1 - (center time / total time) (Zhang, Li, and Han 2020). We did not observe a significant difference in thigmotaxis between mice implanted with a COMBO window and controls at two weeks after surgery (see **Figure R1** below).

**Figure R1.** Animal stress/anxiety was assessed using a measure called “thigmotaxis”, which describes the proportion of time animals spend at the periphery of an open field arena. No significant difference was observed between control animals (N = 7 mice) and those with a COMBO window installed (N = 7 mice). Boxplots represent the median (center line), 25th and 75th percentiles (lower and upper box), and the 1st and 99th percentile (whiskers). One-way ANOVA on ranks with main effect of cranial window.

Additionally, in a separate cohort of animals with a COMBO window installed, we continuously measured the weight of implanted mice after surgery and compared these measurements to age-matched littermate controls. Overall, after an initial decrease in weight during post-op recovery, the animals’ weight stabilized and was similar to that of their littermates within 4-5 days (**Figure S5**). Overall, the similarities between control mice and those implanted with the COMBO window in terms of local neuronal inflammation (GFAP) as well as more global measures such as a behavioral readout of stress and weight measurements, indicate that the COMBO window does not impose a significant burden to the animals after recovery from the surgical procedure. We further have no indication that the impact of this procedure is more pronounced with the COMBO window compared to other similar implants. We have added the continuous weight measurements to the updated **Figure S5** (also below)**,** and provide a concise discussion on this topic in the updated ***Design principles of the COMBO window*** section, as follows:

“We further investigated more global health effects of the COMBO window by tracking the weight of mice after installation. As expected with an invasive surgical procedure, compared to age-matched littermates (N = 4 mice), those mice implanted with the COMBO window (N = 6 mice) exhibited a significant reduction in weight within the first three days after installation (< 10% loss) (**Figure S5a-c**). Nevertheless, the weight of implanted animals returned to pre-surgical and control levels within 4-5 days, and persisted even after attachment of the head plate, suggesting no long-term systemic effects.”

 **Figure S5: Animal weight returns to pre-intervention range after COMBO window installation**

**a-b** The weight of individual animals implanted with a COMBO window (N = 6 mice) (a) and age-matched littermate controls (N = 4 mice) tracked for 10 days after installation (b). **c** Group-level weight values for animals with (blue) and without (black) a COMBO window implanted. Points represent the mean ± s.e.m.. Wilcoxon rank sum test, * p < 0.05, *** p < 0.005, uncorrected.

**R1.3.** In the Discussion, the authors fleetingly mention that compatibility with fMRI could be achieved. Could they expand the current drawings/instructions to include such a setting, at least in terms of the materials needed (without necessarily testing it in fMRI, just to facilitate the future investigations). Also, they should mention the complexity of integrating their COMBO setup with MRI reception coils.

**Response:** Thank you for raising this point. It would be great to expand the COMBO window to even more imaging modalities. Co-first/corresponding author Bradley Edelman has experience combining fUS and fMRI. In his previous work using both modalities, fMRI measurements were performed using loop receive coils (Edelman et al. 2021). This was performed using a rudimentary implant and was only compatible with a 2 cm loop coil, which exhibited relatively low SNR/tSNR. Nevertheless, this previous version was made from a similar material as the COMBO window, also created with a FormLabs SLA 3D printer. As this previous work shows in Supplementary Figure S1D, this material does not induce any morphological distortions or susceptibility artifacts in the raw EPI images, and we thus expect similar results with the COMBO window. Nevertheless, the COMBO window is a much more streamlined design than the previous implant. Following the reviewer’s advice, we have designed an additional “bilateral” version that is physically compatible with both 1 cm loop coils and CryoProbes (**updated File S10**). This design is compatible with ear-bar head fixation, which is common for many MRI contexts, and we have included a new head plate that can be attached to the implant for head fixation outside of the scanner for multimodal imaging of the same animal (**updated File S12**). We have also included schematics in the updated **Figure S2** illustrating how the bilateral version of the COMBO window fits next to these receive coils. We have also expanded on our message regarding fMRI compatibility in the updated ***Design principles of the COMBO window*** sections, as follows:

“Finally, we also provide a separate version that is compatible with magnetic resonance (MR) receive coils (**Figure S2**). With this version, animals can be scanned with MRI before attaching the metallic head-plate using a standard ear bar head fixation. While we did not extensively evaluate this version for potential MR artifacts in the current work, previous studies with implants of a similar material did not significantly distort MR images (Edelman et al. 2021).”

 **Figure S2: The COMBO window is compatible with high-resolution MR receive coils**

**a** Whole-brain implant version (“bilateral”) designed for magnetic resonance imaging (MRI) compatibility. Head fixation can be performed using ear-bars in the scanner using this version. **b** CAD design of a head-plate compatible with the “bilateral” design that can be used outside of the scanner, if desired. **c-d** Schematics of the bilateral implant alongside a standard 1 cm loop coil (c) and a CryoProbe (d). As with any implant, air bubbles should be minimized when installing the implant and dental cement to avoid MR-related artifacts.

**R1.4.** The authors might want to mention that this setup could also benefit studies of resting-state activity and perhaps e.g. cite e.g. (Ma et al, Resting-state hemodynamics are spatiotemporally coupled to synchronized and symmetric neural activity in excitatory neurons, PNAS 2016) and/or similar papers. In fact, an MR compatible setup could greatly benefit the understanding of resting-state brain modes (e.g. Cabral et al Nature Communications 2023) via concurrent fUS or calcium imaging.

**Response:** We thank the reviewer for this highly relevant suggestion. We have added a brief commentary in the updated ***Discussion*** section to highlight how the multimodal capability of the COMBO window can benefit resting state studies to explore the neural basis of large-scale brain modes and their interactions. Please see the text below:

“As such, the multimodal nature of the COMBO window could also be exploited in studies exploring whole-brain networks using resting-state functional connectivity (Bertolo et al. 2023). In this context, the combination of multiple techniques with different neuronal readouts, such as calcium imaging, fMRI, fUS and electrophysiology, potentially even in the same animals could help to extract complementary aspects of brain networks and their organizing principles across scales (Cabral, Fernandes, and Shemesh 2023; Ma et al. 2016). In fact, as we demonstrate in the current work, such brain networks may be further dissected by exploiting the COMBO window and its compatibility with optogenetic stimulations.”

**R1.5.** The COMBO window fits mice of the bl6 strain. Would modifications be needed for other widely-used strains?

**Response:** We appreciate this important question, which we did not systematically test. However, we expect no issue fitting this implant to other widely-used mouse strains for two reasons. First, we have experience in the lab using CH3 mice and have not encountered any issues using the COMBO window with this mouse line. Second, a recent study concluded that skull measurements across strains are generally conserved and that no major morphological differences exist (Gulner, Navabi, and Kodandaramaiah 2022). We have added a note regarding this point in the updated ***Design principles of the COMBO window*** section, as follows:

“We have successfully tested the COMBO window on both C57BL/6 and CH3 mice. As skull morphology is similar across mouse strains (Gulner, Navabi, and Kodandaramaiah 2022), we expect no issues also installing the COMBO window on other mouse lines.”

**Reviewer 2**

The article designs and characterizes a chronic cranial window implantation compatible for multiple techniques; optical and ultrasound imaging, fiber implantation (for optogenetics) and electrophysiology in mice. The design builds and extends nicely on previous work using PMP windows for combined ultrasound and optical imaging, and now provides a nice framework for incorporating fiber implantations and optogenetics. Furthermore, they show that their prep allows for monitoring of orofacial movements and behavior, with no noticeable impact of the implant. The data and methodology is nicely presented, and the experiments are well done and technically sound. Overall this is a nice article that should be published, I just have some minor comments which should be considered prior to acceptance.

**Response:** We highly appreciate the reviewer’s positive outlook on our current manuscript.

**R2.1.** Methods on P19: I would suggest adding a brief description on how the 1024 element probe is connected and operated with the 256 channel scanner (It is described in the reference, but I think should also be briefly described in the article).

**Response:** We thank the reviewer for the careful assessment of the methods section. We have expanded the fUS acquisition details by adding the reviewer’s suggestion in the updated ***fUS acquisition*** methods section, as follows:

“A 4x multiplexer was used to connect the 1024 channel probe to the 256-channel system, with the beamforming and sequences adapted accordingly.”

**R2.2.** Fig 3b, They show averaged traces across multiple mice (46 sessions), could they show what the traces look like from individual trials to give a sense of the feasibility of interpreting single trial recordings?

**Response:** The reviewer raises an interesting point as to what extent fUS, a blood volume-dependent measure, is consistent at the single-trial level. To illustrate the variability of the response, we provide in the updated **Figure S7a-b** the fUS traces from the superior colliculus for all trials in both the awake and anesthetized datasets. As can be seen from these plots, we observed robust visual-evoked activity at the single trial level, even in the awake conditions when variability is increased due to fluctuating behavioral states. **Figure R2** in our response to comment R2.3 also shows an example of an individual trace for a high motion session before and after motion artifact removal, which illustrates that every visual stimulus can be detected even in these high-movement conditions.

In addition to adding these plots to **Figure S7**, we have also added a concise comment about this finding in the updated ***Brain-wide fUS imaging through the COMBO window*** section, as follows:

“As expected, the fUS signal within these regions correlated with the visual block stimulus (rVIS = 0.68, rSC = 0.71) (**Figure 3b**) and the activity was robust at the level of individual trials and sessions, across animals (**Figure S7a**).”

**Figure S7: The COMBO window enables chronic brain-wide acquisition of fUS data**

**a-b** Single-trial fUS traces from the SC plotted for each awake (**a**, N = 4 mice, n = 46 sessions) and anesthetized (**b**, N = 5 mice, n = 32 sessions) recording session. The arrows indicate the direction of the drifting grating visual stimulus. **c** fUS signal in the superior colliculi (SC) and primary visual cortex (VISp) of anesthetized mice covaries in response to drifting gratings in all four cardinal directions (N = 5 mice, n = 32 sessions). The dark black line and light gray shaded area represent the mean ± s.e.m., respectively, across sessions. **d** GLM results from 8 sessions of a single mouse recorded 7 – 9 weeks after being implanted with a COMBO window overlaid on the Allen Brain Atlas. Only voxels with an average T-score > 2 are displayed.

**R2.3.** Movement and measurements of fUS signals.  In fig 4b the authors show the displacement (in x and y) that occurs during locomotion. It would be informative to provide more information or discussion on the reliability of fUS signals during animal movement.

**Response:** We share the interest of the reviewer concerning the reliability of the fUS signal during animal movement. Doppler imaging is sensitive to animal motion and it is currently difficult to perfectly correct for this. Our group and others have reported that some movements of the mouse can create burst artifacts (i.e. large increase in the power doppler signal across the whole image) because tissue motion is not well eliminated through spatiotemporal filtering of the compound images that form a single power Doppler image. However, these burst artifacts are generally transient and shorter than natural hemodynamic changes. We chose to use a previously validated and straightforward technique called “scrubbing” (Brunner et al. 2021) to remove these sudden movement-related artifacts. On top of this, we added an extra processing step to remove lower frequency, global changes that are associated with noise linked to animal movement. Precisely, we performed a spatiotemporal decomposition of the fUS data from the whole session (3D fUS x time) and removed the first principal component. We provide evidence that our motion artifact removal processing works well in the current context based on expected, ground-truth activated regions from the visual system. In **Figure R2** below, we provide an example fUS trace from the superior colliculus before and after motion artifact removal during awake visual stimulation to illustrate the effectiveness of this approach. Note that we chose an example session particularly affected by motion to better illustrate the effectiveness of the method.

We believe that the best way to illustrate the reliability of the fUS signal during animal movement is to compare the raster plots of evoked activity from the same region and same animals in both awake and anesthetized conditions (**Figure S7a-b**). These raster plots convey the increased variability associated with animal movements even after artifact removal, as well as the fact that we can still reliably detect the visual activity for each stimulus.

Moreover, we discuss motion artifacts in detail in the updated ***Discussion*** section, as follows:

“With large cranial openings, the concept of motion-induced artifacts becomes important as the surrounding skull usually stabilizes smaller windows. While our awake fUS data contained higher noise than anesthetized recordings, standard processing steps (e.g. scrubbing and removal of the first principal component, see methods) revealed visual-evoked fUS activity in core visual brain regions. Similarly, optogenetic stimulation of M2 evoked expected patterns of activation that were confirmed at the neuronal level with electrophysiological recordings. These results confirm that motion artifacts can be efficiently removed while preserving the slow hemodynamic response elicited by sensory or optogenetic stimulations, even at the single trial level, albeit with more variability than during anesthesia. In our awake two-photon recordings, we also observed increased framewise displacement during periods of locomotion, however, image stacks were easily recovered using standard registration procedures implemented in open-source analysis toolboxes (Pachitariu et al. 2016). As with any head-fixed experiment, habituation to the fixation is an important step for animal comfort and for minimizing motion-related noise. As explained in our protocol (**Appendix 1**), we find that at least five days of habituation, increasing the duration of head-fixation each day, is sufficient for stable fUS or two-photon recordings using the COMBO window. In the future, a version of the COMBO window that allows for direct attachment of the imaging device (ultrasound probe, miniscope, etc.) to the head-plate (Bergel et al. 2018; 2020) could further reduce the effects of movement on data quality. Such a version could also be integrated in freely-moving contexts and thereby expand the repertoire of behaviors that can be investigated. Nevertheless, the COMBO window provides a flexible, standardized and open-source solution to combine various neural recording and manipulation techniques in awake head-fixed mice.”

**Figure R2. a** fUS signal from the superior colliculus of an awake mouse in response to drifting gratings (dashed line). In this example, we observed a contamination of the fUS signal due to the movement of the animal (measured by the face “video SVD” trace in [c]). **b** After artifact removal (1st PC removed and high motion frame scrubbing) the fUS signal is free of large deflections and expectedly follows the visual stimulation timing. **c** Animal movement extracted from video of the face and anterior part of the body (principal component of the video).

**R2.4.** The authors state that there are no large qualitative differences between locomotion and non-locomotion trials, however, it would be interesting to explore some of the differences and correlate them to the behaviour.  This would also help illustrate the power of the approach.  For example, you might expect the primary sensory cortex (hind/front paw regions), to show larger activation during locomotion due to the paws touching the wheel.

**Response:** We thank the reviewer for the suggestion. In our lab, we are extensively studying spontaneous locomotion and we can report that this behavior triggers widespread activation across the brain that is not very specific to, for example, the primary motor or sensory cortices, as one might expect. This global effect of uninstructed movement has been consistently reported by other groups using wide-field calcium imaging (Musall et al. 2019) or neuropixel recordings (Stringer et al. 2019). This widespread activity is likely driven by neuromodulation and is difficult to separate into individual motor planning/execution and sensory feedback (visual, somatosensory etc.) components, especially using hemodynamic measures of neuronal activity that are rather slow. For the optogenetic activation of M2, the stimulation induces a strong, widespread activation in the brain that seems to dominate the effects of locomotion. This is evident when comparing the trials with and without locomotion at the level of both the whole-brain and individual ROIs (**Figure S8e-f**). As our activity maps are based on the timing of the optogenetic stimulus, the fact that the global increase expected for locomotion is not apparent in only one of the two maps may be due to a timing difference: while the optogenetic stimulation and its induced circuit activity is always time-locked, initiation of locomotion is more variable. This variability in timing could dampen the locomotion-induced neural activity when averaged across many trials. We appreciate the comment of the reviewer that recognizes the power of combining behavior and opto-fUS. Unfortunately, this experiment was not ideal to illustrate that particular point, due to the unspecific effect of locomotion in the brain.

**R2.5.** It would also be nice to show temporal quantitative traces of the fUS signals in a couple of brain regions for both the locomoting and non-locomoting conditions in the supplemental Fig. S6, similar to what is shown in 5f?

**Response:** We thank the reviewer for the suggestion. We have added in **Figure S8f**, analogous to **Figure 5f**, the fUS traces of the striatum and thalamus for locomoting and non-locomoting conditions. We observe no noticeable difference between the two conditions in these two regions in the time-resolved fUS signal. We have updated the ***Multimodal investigation of optogenetic circuit perturbations*** section and **Figure S8** to reflect these results, as follows:

“Overall, we found that the fUS activation patterns were qualitatively similar between the two conditions (**Figure S8e-f**), suggesting that a large portion of the observed brain activity was a direct result of M2 stimulation and not an indirect consequence of the elicited behavior."

**Figure S8: The COMBO window facilitates longitudinal experiments to interrogate neural circuits underlying behavior**

**a** Facial videography was used to monitor animal behavior on a running wheel. Regions-of-interest (ROIs) placed over the whisker pad (violet) and wheel (green) were utilized to capture whisking activity and locomotion, respectively, induced by optogenetic stimulation of the secondary motor cortex (M2). **b** Consecutive trials from the same example session as in Figure 5c showing a robust and reliable increase in whisking in response to optogenetic activation of M2. Each trial was z-scored to a pre-stimulus baseline and rescaled between 0 and 1 for visualization purposes. **c** Region-wise segmented results of the optogenetically-induced fUS activity. Regions are sorted by brain area, and only significantly modulated (correlation between stimulus timing and fUS signal) regions are included (significantly different from zero across sessions, p < 0.01, FDR-corrected). **d** Example coronal slices from the Allen Brain Connectivity Atlas (connectivity.brain-map.org/projection/experiment/287995889). AAV tracings after injection into the M2 (1) show widespread axonal projections from M2 to the striatum, the thalamus and the midbrain (AP +2.30 mm to AP -3.90 mm). Scale bar represents 1 mm. **e** GLM analysis of fUS data in response to optogenetic stimulation of M2 (N = 3 mice, n = 15 sessions). In contrast to Figure 5e, here the trials were separated according to a strong or weak locomotor response (see methods for threshold definition) to optogenetic stimulation. Only the voxels with T-scores significantly different from zero across sessions (p < 0.05, FDR-corrected) are shown. **f** Group-level fUS traces from the striatum (left) and thalamus (right) for trials with and without animal locomotion**.** The dark lines and lighter shaded areas represent the mean ± s.e.m., respectively, across sessions. **g** Pie chart showing the proportion of different cell response types observed in the midbrain (see methods for cell response type definition).

**R2.6.** P14: Compatibility of window with ephys.  Since they need to drill through the window, are these terminal experiments? It would be important to describe this, as this represents a limitation worth discussing. What is the size of the "small perforations"?

**Response:** Thank you for asking about this important feature of the COMBO window. The electrophysiological recordings are not terminal procedures. We perform these recordings after the fUS experiments, but we acquired multiple recordings over a period of several days before euthanasia. Here the electrophysiological recordings were performed under anesthesia, but a similar protocol with multiple successive recordings across days can be followed for awake mice (Steinmetz et al. 2019). No pain is associated with drilling through the PMP film because the skull and skin are already removed and the animals are anesthetized during this procedure. The holes are covered with Kwik-Cast silicone sealant after each recording. Regarding the size of the hole, the perforations in our case were as large as the tip of the drill (0.5 mm), however, most slim silicone probes can theoretically accommodate even smaller perforations. We have added various details regarding the electrophysiological recordings in both the updated ***Multimodal investigation of optogenetic circuit perturbations*** section and the ***Electrophysiology recordings*** method section, as follows:

“For this, we drilled small perforations (0.5 mm diameter) in the film and lowered 32-channel silicon probes into the midbrain at coordinates where we previously observed strong fUS activation (AP: -4.00 mm, DV: -2.20 to -3.60 mm, ML: ±1.0 mm) (**Figure 5e, g-h**), and optogenetically activated M2 at 5 Hz.”

“Small perforations in the COMBO window film above the midbrain (AP: 4.00 mm, ML: 1.00) were created using a drill (0.5 mm diameter), and the probe was lowered into the midbrain at a speed of 2 µm/s. Data was acquired at three different depths (DV: -2.20 mm, -2.60 mm and -3.60 mm below the brain surface) in both hemispheres during separate recordings. Concurrent optogenetic stimulation and electrophysiological recordings were performed 10 min after reaching the desired depth. In cases where the separate recordings were performed on different days, the perforations in the window were sealed with Kwik-Cast silicone sealant. CM-Dil (Thermo Fisher, CellTracker) was applied to the probe prior to brain insertion to allow post-hoc confirmation of the recording site.”

**R2.7.** A couple of control experiments for the light stimulation alone (in ChR2 negative mice) would be appreciated.

**Response:** Thank you for suggesting this important control as light-induced effects have been previously observed with hemodynamic signals. To address this, we have performed a control experiment where we bilaterally injected an AAV9-CamkIIa-EGFP virus (non-ChR2) into M2 in three animals, and performed the same optogenetic protocol as previously reported for ChR2-positive animals in both awake (N = 3 mice, n = 12 sessions) and anesthetized (N = 3 mice, n = 16 sessions) contexts. We specifically decided to acquire fUS recordings in the mice under anesthesia to maximize the possibility to detect potential light-induced activity as the signal-to-noise ratio is higher than in awake conditions. Overall, we found that the previously observed fUS effects illustrated in **Figure 5** were not present in these control animals for either awake or anesthetized contexts, confirming that they were in fact induced via optogenetic activation of M2 and not light itself. Similarly, we observed no effect of stimulation on whisking or locomotion in control animals in the awake context. The data from these new experiments is uploaded to our repository. To clearly convey this message, we have added the behavior quantification and the ROI fUS time series for the control animals to **Figure 5**. We also report in the new **Figure S9** un-thresholded T-maps for the awake ChR2 animals alongside the awake and anesthetized control animals. We have added these results throughout the text in the updated ***Multimodal investigation of optogenetic circuit perturbations*** section as shown below.

“To control for potential light-induced effects, we also injected a non-ChR2 carrying virus (AAV9-CaMKIIa-EGFP) into M2 of wild-type C57BL/6 mice (N = 3 mice) and applied the same stimulation protocol. In these control mice, we did not observe a noticeable effect for locomotion (peak z-score: 0.3 ± 0.18) or whisking (peak z-score: 0.14 ± 0.13) (**Figure 5d**).”

“Additionally, as light-induced vasodilation has been previously reported (Rungta et al. 2017; Schmid et al. 2017; Christie et al. 2013), we examined in detail the responses in awake “non ChR2” mice (N = 3 mice, n = 12 sessions) and recorded additional sessions under anesthesia (N = 3 mice, n= 14 sessions) to maximize the detectability of potential light effects. Analogous to the behavioral analysis, we did not find any significant evoked neural activation in response to stimulation in control mice (“non-ChR2”) for either awake or anesthetized contexts (**Figure 5f**, **Figure S9**), demonstrating that the observed neural responses are due to optogenetic M2 activation. These results together demonstrate that the COMBO window enables the observation of a large volume of the brain during bilateral optogenetic stimulation in behaving animals.”

**Figure 5: Multimodal investigation of optogenetic circuit perturbations**

**a** Schematic representation of simultaneous fUS imaging and optogenetic stimulation (473 nm) of secondary motor cortex (M2) in mice with the “anterior” version of the COMBO window on a running wheel. **b** Before window implantation, AAV9-hChR2(E123A)-EYFP was injected bilaterally into M2, resulting in robust expression of hChR2(E123A)-EYFP (EYFP in green, and DAPI in magenta). Scale bar represents 1 mm. **c-d** Behavioral parameters were assessed by quantifying motion energy changes from wheel and whisker regions-of-interest (ROIs). **c** Consecutive trials from one session after applying a z-score transformation to the pre-stimulus baseline. Traces are rescaled between zero and one for visualization purposes. **d** Optogenetic activation of M2 results in a robust increase in locomotion and whisking (locomotion: 8.25 ± 1.89 peak z-score; whisking: 1.86 ± 0.40 peak z-scores (mean ± s.e.m.) (N = 5 mice, n = 22 sessions) (left). These effects were absent in non-ChR2 expressing control animals (locomotion: 0.32 ± 0.20 peak z-score; whisking: 0.13 ± 0.14 peak z-score) (N = 3 mice, n = 14 session) (right). **e** Average T-maps are overlaid on the Allen Brain Atlas at the indicated positions (AP distance from bregma). Voxels with a T-score significantly different from zero across sessions (p < 0.05, FDR corrected) are displayed in color (N = 3 mice, n = 16 sessions). White square boxes represent ROIs used to extract fUS time series data in **f** and **h**. **f** Group-level fUS traces from the striatum and thalamus for ChR2-expressing animals (green) and non-ChR2 expressing control animals (gray). **g** Electrophysiological recordings were performed during optogenetic stimulation of M2 to verify the fUS signal. Silicon probes successfully targeted the midbrain (CM-Dil dye in red and DAPI in cyan), where strong optogenetically-induced fUS activation was observed. Scale bar represents 1 mm. **h** The mean normalized firing rate across all recorded cells in the midbrain (bottom) compared to the average fUS signal (top) of ROI 3 (midbrain) from **e**. **i** The normalized firing rate of all recorded cells sorted by mean activity during optogenetic stimulation. **j** Raster plots showing all spikes from a given session for three example cells, highlighting the diversity of single-cell responses. For all panels, blue squares indicate the time of optogenetic stimulation. Solid lines and shaded areas represent the mean ± s.e.m. across sessions or cells.

**Figure S9. Blue light stimulation alone does not induce neural activation**

**a** Optogenetic activation of M2 results in widespread activation in ChR2-expressing mice. Average T-scores at indicated positions (distance to Bregma) are shown without thresholding (left, N = 3 mice, n = 16 sessions). Histograms of the same T-scores color-coded by significance (p<0.05, FDR-corrected) (right). **b-c** Same as (a) for animals injected with an AAV9-CamkIIa-EGFP control virus in M2. Low T-scores and no significant voxels (p<0.05, FDR-corrected) are observed for both the awake (N = 3 mice, n = 12 sessions) and anesthetized (N = 3 mice, n = 14 sessions) conditions.

**R2.8.** On this topic, light stimulation itself has been reported to cause local vasodilation and fUS signals (e.g. PMID 28139643). I realize that the authors do not image directly next to the fiber, where this artifact would be potentially expected to occur, but this could at least be briefly mentioned in discussion. This same paper should also be cited in the introduction in a different context, as it was the first study to combine fUS and 2P imaging with PMP windows, as expanded upon here.

**Response:** Thank you for reminding us of this work. We apologize for this omission in the original submission as this is a seminal work for opto-fUS and multimodal imaging with fUS. We have added this citation to the updated ***Introduction*** section, as follows:

“Despite these advantages, such designs have not been tested for acoustic imaging and are still limited in field of view. In parallel, other implants have been proposed to specifically accommodate fUS while being compatible with optical methods (Aydin et al. 2020; Boido et al. 2019; Kılıç et al. 2020; Rungta et al. 2017).”

As described above, our additional results addressing this point experimentally are now presented in **Figure 5 and Figure S9**. We also added new commentary in the updated ***Discussion*** section on this work and how opto-fUS results must be carefully interpreted due to the effect of light on vasodilation. Please find this text below:

“In fact, as we demonstrate in the current work, such brain networks may be further dissected by exploiting the COMBO window and its compatibility with optogenetic stimulations. In this context, however, researchers should exercise caution when interpreting opto-fUS results since local vasodilation has previously been observed with both fUS and fMRI in response to direct light stimulation at intensities as low as 2 mW (Rungta et al. 2017; Schmid et al. 2017; Christie et al. 2013). In the current work, we ensured that no unspecific light effects contaminated our results by repeating the optogenetic stimulations in control animals that only expressed GFP in M2 (non-ChR2) in both awake (same as the ChR2-positive group) and anesthetized states. In the latter, behavior-induced variability and noise in the fUS signal is minimal; in these optimal conditions and with a relatively high light intensity (5-6 mW) we did not observe any significant light-induced activation. We could not image the stimulation site directly and therefore cannot rule out local effects, however, these results suggest that the observed brain-wide activity in response to optogenetic M2 activation in ChR2-expressing animals was not caused by light-induced vasodilation.”

**R2.9.** Typo P16L31, visually visual-evoked

**Response:** We thank the reviewer for the attentive reading of the manuscript and have corrected the mistake.

**Reviewer 3**

This paper introduces the COMBO window - an open-source method for creating 3D printed customized cranial windows that apply to calcium imaging and fUS. This work builds on previous work done for wide field cortical imaging that uses a similar approach to those developed previously. The relative advance here is that the authors provide several different versions of the polymer cranial windows which could be useful in a number of different contexts. There is also an increased coverage in terms of the FOV. The authors also show fUS imaging and calcium imaging. Overall, this is a work that could be useful to a number of labs.

I do have some comments and concerns that should be addressed/ modified before publication.

**Response:** We thank the reviewer for their encouraging comments and positive outlook for how the COMBO window can impact the scientific community.

**R3.1.** A central argument for the development of the COMBO window is that it is uniquely developed for fUS imaging. The authors argue that the previous cranial windows with transparent films (such as the paper cited i.e., Ghanbari et al and other derivatives) are incompatible with fUS imaging (or as stated in the paper ‘have issues’). It is unclear whether the authors tested such implants for fUS imaging, or if there is literature that suggest incompatibility. The only difference between the two designs is the use of the polymer films - Ghanbari et al used PET films, whereas this work uses PMP films.

Is there something specific about PMP that helps with fUS imaging?

Did the authors evaluate the use of PET for fUS imaging? If so, what were the negative results, and how did they compare with PMP films? If there is no such comparative experiment conducted, it is unclear how the authors conclude previous window implants using PET films have an issue.

If there no evidence of issues either from previous studies or any experiments that the authors conducted, then this central argument of this paper needs to be watered down - it is fine to simply state this approach is compatible with fUS and that other approaches may work as well, it just not been tested.

**Response:** We apologize for the lack of clarity and we agree with the reviewer that the comparison with other implants should not be misleading. We did not mean to imply that the previous implants using PET are incompatible with fUS as it has simply not been comprehensively tested. This is of course different for glass implants that cannot work for fUS due to the strong attenuation of ultrasound waves through this material. When choosing a polymer, we chose PMP, which has been previously used in the field of fUS because it has been described as having good acoustic properties (see [link](https://www.goodfellow.com/information/larger-quantities/513/544/polymers/tpx-products) to the manufacturer page). Indeed, it is very likely that PET would work well. More important than the type of polymer, the thickness of the film usually has the largest impact on the attenuation of ultrasound waves. As the reviewer suggested, we have corrected both the ***Introduction*** and ***Design principles of the COMBO window*** sections to highlight that other polymer-based films are likely compatible with fUS. We also now focus more on the specific advantages and features of the COMBO window design, namely the large field-of-view, the compatibility with behavior, and the flexible design for multimodal imaging and optogenetic applications.

***Introduction***:

“The COMBO window aims to combine and expand the advantages of currently available cranial window implants by providing (1) a larger field of view, (2) compatibility with optical and acoustic imaging, (3) integration with local recording and manipulation methods, and (4) unobstructed access to head-fixed behavioral readouts.”

***Design principles of the COMBO window:***

“By testing different thicknesses, we have found that 125 um PMP offers an optimal compromise specifically for the COMBO window between flexibility and durability. PMP was chosen for its good acoustic properties, however it is likely that others polymers (e.g. PET, as used with the See-Shell (Ghanbari et al. 2019)) are also compatible with fUS imaging (G. Kim et al. 2023; Ghanbari et al. 2019).”

**R3.2.** The authors show for a paper that is touting a new kind of cranial window technology, there is very little characterization of the long term effect of the window implantation on the brain. This information will provide potential users context on feasibility of implementation. Two aspects that need to be described more:

The authors state that the COMBO windows provide increased imaging coverage. While the analysis of orofacial behavior is nice, what is more critical is to provide data on success rate of surgical implantation. This will provide potential users important context on the feasibility of the COMBO window approach. Several questions arise:

**R3.2.1.** How many mice were implanted? What is the overall success rate of these surgeries?

**R3.2.2.** What are typical failure points?

**R3.2.3.** How long do these windows last? What are the neuroimmune responses that occur over time?

**R3.2.4.** Cranial window implants are often accompanied by dura thickening, bone regrowth and general occlusion of the FOV. Did the authors encounter these issues? If so these outcomes with n values need to be reported.

**Response:** Thank you for these suggestions. Based on the reviewer’s suggestion, we strengthened the characterization of the implant in the revised manuscript.

First, as we mention in our response to comment R1.2, we now include additional information to better characterize the long-term effects of the COMBO window. To complement our initial analysis on the local inflammatory response in the brain and the freely moving behavior, we used a separate cohort of animals to examine the weight of animals implanted with a COMBO window and their age-matched littermates. As expected for such an invasive procedure, we observed a significant decrease in weight at 2-3 days following the surgery (still within an acceptable range of < 10% loss). We further observed that the weight of COMBO animals quickly returned within five days to a weight range similar to pre-intervention levels and to time-matched controls. These results and a description thereof have been added to the updated **Figure S5** and the ***Design principles of the COMBO window*** section, as follows:

“We further investigated more global health effects of the COMBO window by tracking the weight of mice after installation. As expected with an invasive surgical procedure, compared to age-matched littermates (N = 4 mice), those mice implanted with the COMBO window (N = 6 mice) exhibited a significant reduction in weight within the first three days after installation (< 10% loss) (**Figure S5a-c**). Nevertheless, the weight of implanted animals returned to pre-surgical and control levels within 4-5 days, and persisted even after attachment of the head plate, suggesting no long-term systemic effects.”

 **Figure S5: Animal weight returns to pre-intervention range after COMBO window installation**

**a-b** The weight of individual animals implanted with a COMBO window (N = 6 mice) (a) and age-matched littermate controls (N = 4 mice) tracked for 10 days after installation (b). **c** Group-level weight values for animals with (blue) and without (black) a COMBO window installed. Points represent the mean ± s.e.m.. Wilcoxon rank sum test, * p < 0.05, *** p < 0.005, uncorrected.

We also re-analyzed our open field data to examine the time spent in the center of the field compared to the spent next to the walls (a measure called “thigmotaxis”), which is a standard test for anxiety/stress (stressed animals explore the center area less (Zhang, Li, and Han 2020)). We did not find any significant difference between the non-implanted and COMBO animals. These two tests could not detect any long-term impact of the implantation on the well-being of mice after the post-surgery recovery period.

**Figure R1.** Animal stress/anxiety was assessed using a measure called “thigmotaxis”, which describes the proportion of time animals spend at the periphery of an open field arena. No significant difference was observed between control animals (N = 7 mice) and those with a COMBO window installed (N = 7 mice). Boxplots represent the median (center line), 25th and 75th percentiles (lower and upper box), and the 1st and 99th percentile (whiskers). One-way ANOVA on ranks with main effect of cranial window.

Additionally, to address the important questions in comments R3.2.1. – 3.2.4. we report in the text the overall surgical success rate, occurrence of issues occluding the cranial window, and typical failure points. We used the COMBO window extensively in the lab and can report a success rate of 82% from the 65 mice implanted by 4 surgeons. To address the occlusion point, we focused on a subset of 37 mice in which a COMBO window was successfully installed and that were consistently scanned with fUS for eight weeks or more. In this cohort, we found a significant degradation of the fUS imaging quality due to dura thickening in 11% of mice (4/37). The other mice exhibited consistent imaging quality at the vascular (Doppler) and functional (visual responses) level (see **Figure 2 and Figure S7d**). Optical transparency was also confirmed with multi-region two-photon calcium imaging at 10 weeks in 3/3 GCaMP mice (**Figure 4**). Our animal licenses imposed a limit of 63 days, and therefore we could not evaluate the image quality beyond that time.

We have updated the text in the ***Design principles of the COMBO window*** section to describe this:

“The overall success rate of COMBO window installation across users in our lab was 82% (4 surgeons, 53/65 mice). The success of this procedure is based on strict animal welfare criteria in the post-operative phase. Failure points involved welfare endpoints being reached during the recovery period or loss of the implant due to insufficient dental cement adhesion; such cases typically occurred within one week of surgery. A subset of the mice with successful COMBO window installation was consistently scanned with fUS for eight weeks or more. In only 11% (4/37) of the mice did we observe the emergence of window occlusions that caused an obvious reduction of fUS imaging quality. We could not evaluate the lifetime of windows beyond the limit imposed by our ethics protocols (maximum 63 days), however, many of the experiments performed in the current work and described in later sections took place as late as ten weeks after implant installation with consistent imaging quality for acoustic and optical imaging.”

**R3.3.** Unclear why the authors do the analyses of free behavior - as the imaging is anyway done in headfixed animals...

**Response:** Thank you for asking for clarification on this. We think it is important to assess the impact of the COMBO window on freely moving behavior to evaluate if it impacts the well-being of the mice. Such effects could distort the results of head-fixed behavioral experiments even though the effect would not be directly quantifiable in that context. In particular, we wanted to ensure that the additional weight of the implant does not affect the animals’ locomotion as impaired locomotion and/or mobility can negatively affect the animals’ quality of life and induce stress. We performed an open-field foraging task to assess this effect, and found that animal locomotion is in fact preserved. These results suggest that the COMBO window does not introduce behavioral biases and that the animals are not burdened by the implant in the home cage. We have updated our justification for this experiment in the ***The COMBO window preserves freely moving behavior and facial expressions*** section, as follows:

“We next characterized the impact of the COMBO window on various aspects of mouse behavior. Behavioral effects due to the added weight of the implant may not be apparent in head-fixed experiments, but could significantly affect an animal’s locomotion in freely moving contexts (e.g. the home cage), and negatively affect their quality of life and induce stress over time (Hobbiesiefken et al. 2021). Therefore, we assessed the effects of head-plate weight on natural behavior during an open-field free foraging task (**Figure 2a**) (Zong et al. 2022).”

**R3.4.** With the increased coverage, what is the effect of motion artifacts if any on imaging/ ephys. The authors correct for lateral displacement in x and y, but there is no comment on the z displacement.

**Response:** We thank the reviewer for the comment on motion artifacts. As we performed single plane two-photon imaging, no correction of z-displacement was performed, however, only cells that were stable across the entire time of the recording were included in the analysis. We have added a brief description of our criteria for stability in the updated ***Two-photon microscopy analysis*** methods section, as follows:

“Briefly, we first imposed a strict skewness threshold of 2 and then manually discarded cells if the corresponding time series exhibited sudden jumps representative of significant z-displacements.”

For fUS imaging, motion artifacts are complex and there is currently no standardized approach in the field to address them. Nevertheless, as we mention in our response to comment R2.3, we implemented a previously validated scrubbing technique (Brunner et al. 2021), combined with the removal of the 1st principal component of the fUS time series to address motion artifacts. The visual dataset also serves as a “ground truth” context to validate this approach as the response to visual stimuli with fUS is well characterized (Macé et al. 2018; Gesnik et al. 2017). We also addressed this point experimentally by comparing visual responses in the awake context to those of the anesthetized context when the motion-related noise is much lower. We found that we could effectively map the activity in core visual regions in awake mice using the COMBO window (see also response to point comment R2.2). We have added clarification on this to the updated ***fUS preprocessing*** methods section and the ***Discussion***, as follows:

**fUS preprocessing:**

“Lastly, to remove unspecified movement artifacts during awake experiments, we removed the first principal component from the spatial-temporal decomposition of the entire time series. In addition, we implemented additional motion artifact rejection according to a previously validated approach (Brunner et al. 2021). For this, we removed motion artifact time points by interpolating those that were above a voxel-specific threshold, defined as the median + absolute deviation of signal * 4.44.”

**Discussion:**

“With large cranial openings, the concept of motion-induced artifacts becomes important as the surrounding skull usually stabilizes smaller windows. While our awake fUS data contained higher noise than anesthetized recordings, standard processing steps (e.g. scrubbing and removal of the first principal component, see methods) revealed visual-evoked fUS activity in core visual brain regions. Similarly, optogenetic stimulation of M2 evoked expected patterns of activation that were confirmed at the neuronal level with electrophysiological recordings. These results confirm that motion artifacts can be efficiently removed while preserving the slow hemodynamic response elicited by sensory or optogenetic stimulations, even at the single trial level, albeit with more variability than during anesthesia. In our awake two-photon recordings, we also observed increased framewise displacement during periods of locomotion, however, image stacks were easily recovered using standard registration procedures implemented in open-source analysis toolboxes (Pachitariu et al. 2016). As with any head-fixed experiment, habituation to the fixation is an important step for animal comfort and for minimizing motion-related noise. As explained in our protocol (**Appendix 1**), we find that at least five days of habituation, increasing the duration of head-fixation each day, is sufficient for stable fUS or two-photon recordings using the COMBO window. In the future, a version of the COMBO window that allows for direct attachment of the imaging device (ultrasound probe, miniscope, etc.) to the head-plate (Bergel et al. 2018; 2020) could further reduce the effects of movement on data quality. Such a version could also be integrated in freely-moving contexts and thereby expand the repertoire of behaviors that can be investigated. Nevertheless, the COMBO window provides a flexible, standardized and open-source solution to combine various neural recording and manipulation techniques in awake head-fixed mice.”

Finally, in the context of the electrophysiological recordings, motion artifacts were negligible in the current work as the experiments were performed under anesthesia.

**R3.5.** Some of the experiments are under powered - n=2 for optogenetic experiments, n =2 electrophysiology experiments. How well does the approach work?

**Response:** Thank you for raising this point regarding the animal numbers used in the current work. Here, we focussed on a proof of concept opto-fUS demonstration, and simply wanted to demonstrate the detectability of brain regions significantly activated by the optogenetic stimulation. As such, the GLM containing a regressor contrasting the ON and OFF stimulation periods illustrates significant activation at the voxel level. Following the reviewer’s suggestion, we have now performed additional experiments on one more ChR2 animal (n = 4 awake sessions) and three non-ChR2 control animals (n = 12 awake sessions in total) to increase the N specifically for the opto-fUS data and to match animal numbers in both groups (also with our other experiments, i.e. facial expressions, two-photon). The new data has been uploaded on our online repository and we have added the results from this new opto-fUS cohort to the updated **Figure 5,** **Figure S8,** and **Figure S9**. We have also incorporated the relevant changes in the text throughout the ***Multimodal investigation of optogenetic circuit perturbations*** section***.***

Regarding the procedure itself, the window implantation for the opto-fUS approach works as well as the implantation of the standard “flat” or “cup” versions, however, additional variables common to optogenetic experiments must also be considered. These variables include virus expression, fiber placement, and fiber loss; however, we did not specifically lose any animals due to these reasons. Please also see our detailed description of surgical success in our response to comment R3.2.

**Figure 5: Multimodal investigation of optogenetic circuit perturbations**

**a** Schematic representation of simultaneous fUS imaging and optogenetic stimulation (473 nm) of secondary motor cortex (M2) in mice with the “anterior” version of the COMBO window on a running wheel. **b** Before window implantation, AAV9-hChR2(E123A)-EYFP was injected bilaterally into M2, resulting in robust expression of hChR2(E123A)-EYFP (EYFP in green, and DAPI in magenta). Scale bar represents 1 mm. **c-d** Behavioral parameters were assessed by quantifying motion energy changes from wheel and whisker regions-of-interest (ROIs). **c** Consecutive trials from one session after applying a z-score transformation to the pre-stimulus baseline. Traces are rescaled between zero and one for visualization purposes. **d** Optogenetic activation of M2 results in a robust increase in locomotion and whisking (locomotion: 8.25 ± 1.89 peak z-score; whisking: 1.86 ± 0.40 peak z-scores (mean ± s.e.m.) (N = 5 mice, n = 22 sessions) (left). These effects were absent in non-ChR2 expressing control animals (locomotion: 0.32 ± 0.20 peak z-score; whisking: 0.13 ± 0.14 peak z-score) (N = 3 mice, n = 14 session) (right). **e** Average T-maps are overlaid on the Allen Brain Atlas at the indicated positions (AP distance from bregma). Voxels with a T-score significantly different from zero across sessions (p < 0.05, FDR corrected) are displayed in color (N = 3 mice, n = 16 sessions). White square boxes represent ROIs used to extract fUS time series data in **f** and **h**. **f** Group-level fUS traces from the striatum and thalamus for ChR2-expressing animals (green) and non-ChR2 expressing control animals (gray). **g** Electrophysiological recordings were performed during optogenetic stimulation of M2 to verify the fUS signal. Silicon probes successfully targeted the midbrain (CM-Dil dye in red and DAPI in cyan), where strong optogenetically-induced fUS activation was observed. Scale bar represents 1 mm. **h** The mean normalized firing rate across all recorded cells in the midbrain (bottom) compared to the average fUS signal (top) of ROI 3 (midbrain) from **e**. **i** The normalized firing rate of all recorded cells sorted by mean activity during optogenetic stimulation. **j** Raster plots showing all spikes from a given session for three example cells, highlighting the diversity of single-cell responses. For all panels, blue squares indicate the time of optogenetic stimulation. Solid lines and shaded areas represent the mean ± s.e.m. across sessions or cells.

**Figure S8: The COMBO window facilitates longitudinal experiments to interrogate neural circuits underlying behavior**

**a** Facial videography was used to monitor animal behavior on a running wheel. Regions-of-interest (ROIs) placed over the whisker pad (violet) and wheel (green) were utilized to capture whisking activity and locomotion, respectively, induced by optogenetic stimulation of the secondary motor cortex (M2). **b** Consecutive trials from the same example session as in Figure 5c showing a robust and reliable increase in whisking in response to optogenetic activation of M2. Each trial was z-scored to a pre-stimulus baseline and rescaled between 0 and 1 for visualization purposes. **c** Region-wise segmented results of the optogenetically-induced fUS activity. Regions are sorted by brain area, and only significantly modulated (correlation between stimulus timing and fUS signal) regions are included (significantly different from zero across sessions, p < 0.01, FDR-corrected). **d** Example coronal slices from the Allen Brain Connectivity Atlas (connectivity.brain-map.org/projection/experiment/287995889). AAV tracings after injection into the M2 (1) show widespread axonal projections from M2 to the striatum, the thalamus and the midbrain (AP +2.30 mm to AP -3.90 mm). Scale bar represents 1 mm. **e** GLM analysis of fUS data in response to optogenetic stimulation of M2 (N = 3 mice, n = 15 sessions). In contrast to Figure 5e, here the trials were separated according to a strong or weak locomotor response (see methods for threshold definition) to optogenetic stimulation. Only the voxels with T-scores significantly different from zero across sessions (p < 0.05, FDR-corrected) are shown. **f** Group-level fUS traces from the striatum (left) and thalamus (right) for trials with and without animal locomotion**.** The dark lines and lighter shaded areas represent the mean ± s.e.m., respectively, across sessions. **g** Pie chart showing the proportion of different cell response types observed in the midbrain (see methods for cell response type definition).

**Figure S9. Blue light stimulation alone does not induce neural activation**

**a** Optogenetic activation of M2 results in widespread activation in ChR2-expressing mice. Average T-scores at indicated positions (distance to Bregma) are shown without thresholding (left, N = 3 mice, n = 16 sessions). Histograms of the same T-scores color-coded by significance (p<0.05, FDR-corrected) (right). **b-c** Same as (a) for animals injected with an AAV9-CamkIIa-EGFP control virus in M2. Low T-scores and no significant voxels (p<0.05, FDR-corrected) are observed for both the awake (N = 3 mice, n = 12 sessions) and anesthetized (N = 3 mice, n = 14 sessions) conditions.

**R3.6.** For calcium imaging, the author developed a flat version. How large of a coverslip was used in this case? One of the issues is that implanting something like this over a very large area will results in several tens of mm^3 of brain tissue being displaced. While the experiments were done on 3 animals, there is no data provided on histology post implantation.

**Response:** We apologize for the lack of clarity in our description of the various implant versions. The “flat” version name simply refers to the absence of the upward protruding cup (intended to hold ultrasound gel), with all other aspects of the implant being exactly the same as the “cup” version intended for use with fUS. This simply allows for a closer working distance of the objective. Thus, in the flat version (and all versions) the same plastic film is fitted to the curvature of the skull and brain and no glass coverslip is used. The curvature of the implant and plastic film was specifically designed to accommodate the curvature of the brain to avoid anatomical distortions. Furthermore, as this curvature is the same for all versions of the implant, we feel that the immunohistochemical analysis of inflammation (measured using GFAP) applies universally to all versions of the COMBO window. In this regard, we describe in the ***Design principles of the COMBO window*** section and **Figure 1f** that there is no significant inflammatory response or morphological distortion between control animals and those implanted with the COMBO window. We have added concise clarifications on the description of the flat version at various locations in the manuscript, as follows:

**Design principles of the COMBO window:**

“Note that fUS can also be performed through the flat version and that only the external rim differs between the two versions (i.e. the film itself is not flat, as explained below).”

**The COMBO window enables multisite two-photon imaging during behavior:**

“To assess the ability to capture single-cell activity through the COMBO window using optical imaging techniques, we created the “flat” version by removing the upward protruding cup (the film still shaped to the curvature of the brain), which allows closer positioning of the objective towards the brain surface (working distance of objective: 2 mm).”

**References:**

Brunner, Clément, Micheline Grillet, Alan Urban, Botond Roska, Gabriel Montaldo, and Emilie Macé. 2021. “Whole-Brain Functional Ultrasound Imaging in Awake Head-Fixed Mice.” *Nature Protocols* 16 (7): 3547–71. https://doi.org/10.1038/s41596-021-00548-8.

Edelman, Bradley Jay, Giovanna D. Ielacqua, Russell W. Chan, Mazen Asaad, Mankin Choy, and Jin Hyung Lee. 2021. “High-Sensitivity Detection of Optogenetically-Induced Neural Activity with Functional Ultrasound Imaging.” *NeuroImage* 242 (November): 118434. https://doi.org/10.1016/j.neuroimage.2021.118434.

Gesnik, Marc, Kevin Blaize, Thomas Deffieux, Jean-Luc Gennisson, José-Alain Sahel, Mathias Fink, Serge Picaud, and Mickaël Tanter. 2017. “3D Functional Ultrasound Imaging of the Cerebral Visual System in Rodents.” *NeuroImage* 149 (April): 267–74. https://doi.org/10.1016/j.neuroimage.2017.01.071.

Gulner, Beatrice R., Zahra S. Navabi, and Suhasa B. Kodandaramaiah. 2022. “3D Morphometric Analysis of Mouse Skulls Using Microcomputed Tomography and Computer Vision.” bioRxiv. https://doi.org/10.1101/2022.10.26.513830.

Macé, Émilie, Gabriel Montaldo, Stuart Trenholm, Cameron Cowan, Alexandra Brignall, Alan Urban, and Botond Roska. 2018. “Whole-Brain Functional Ultrasound Imaging Reveals Brain Modules for Visuomotor Integration.” *Neuron* 100 (5): 1241-1251.e7. https://doi.org/10.1016/j.neuron.2018.11.031.

Musall, Simon, Matthew T Kaufman, Ashley L Juavinett, Steven Gluf, and Anne K Churchland. 2019. “Single-Trial Neural Dynamics Are Dominated by Richly Varied Movements.” *Nature Neuroscience* 22 (10): 1677–86. https://doi.org/10.1038/s41593-019-0502-4.

Steinmetz, Nicholas A., Peter Zatka-Haas, Matteo Carandini, and Kenneth D. Harris. 2019. “Distributed Coding of Choice, Action, and Engagement across the Mouse Brain.” *Nature* 576 (7786): 266–73. https://doi.org/10.1038/s41586-019-1787-x.

Stringer, Carsen, Marius Pachitariu, Nicholas Steinmetz, Charu Bai Reddy, Matteo Carandini, and Kenneth D. Harris. 2019. “Spontaneous Behaviors Drive Multidimensional, Brainwide Activity.” *Science* 364 (6437): 255. https://doi.org/10.1126/science.aav7893.

Zhang, Chen, Haiwen Li, and Renzhi Han. 2020. “An Open-Source Video Tracking System for Mouse Locomotor Activity Analysis.” *BMC Research Notes* 13 (1): 1–6. https://doi.org/10.1186/S13104-020-4916-6/FIGURES/3.
