# Supplementary material for: The COMBO window: A chronic cranial implant for multiscale circuit interrogation in mice
Source: PLoS Biol. 2024 Jun 3;22(6):e3002664. doi: 10.1371/journal.pbio.3002664 (PMC11185485; doi:10.1371/journal.pbio.3002664)
Supplement: Attachment — Submitted filename: EdelmanSiegenthaler2023_ResponseToEditor_R2.docx [file pbio.3002664.s057.docx]

PBIOLOGY-D-23-02796R2 Author Response

**(A)** In the animal ethics statement in the Methods section, I would be grateful if you could please specify that the mouse studies were specifically reviewed and approved by an IACUC or animal ethics committee. Please include the full name of the IACUC/ethics committee and include an approval number.

**Response:** We have updated the methods section and added an “Ethics Statement”. Please find this information below:

“**Ethics statement**

All experiments were carried out in compliance with institutional guidelines of the Max Planck Society and of the local government (Regierung von Oberbayern) under license numbers 55.2-2532.Vet_02-21-190, 55.2-2532.Vet_02-21-14, 55.2-2532.Vet_02-20-123..”

**(B)** You may be aware of the PLOS Data Policy, which requires that all data be made available without restriction: <http://journals.plos.org/plosbiology/s/data-availability>. For more information, please also see this editorial: <http://dx.doi.org/10.1371/journal.pbio.1001797>

Note that we do not require all raw data. Rather, we ask that all individual quantitative observations that underlie the data summarized in the figures and results of your paper be made available in one of the following forms:

-Supplementary files (e.g., excel). Please ensure that all data files are uploaded as 'Supporting Information' and are invariably referred to (in the manuscript, figure legends, and the Description field when uploading your files) using the following format verbatim: S1 Data, S2 Data, etc. Multiple panels of a single or even several figures can be included as multiple sheets in one excel file that is saved using exactly the following convention: S1_Data.xlsx (using an underscore).

-Deposition in a publicly available repository. Please also provide the accession code or a reviewer link so that we may view your data before publication.

Regardless of the method selected, please ensure that you provide the individual numerical values that underlie the summary data displayed in the following figure panels as they are essential for readers to assess your analysis and to reproduce it:

Figure 1F, 2C-F, 2I-J, 3B, 3D-E, 4B-C, 4E, 5C-D, 5F, 5H-J, S5A-C, S6A-C, S7A-C, S8B-C, S8F, S9A-C

NOTE: the numerical data provided should include all replicates AND the way in which the plotted mean and errors were derived (it should not present only the mean/average values).

**Response:** We have included Supplementary Data Files 1-10 containing all of the underlying data, including all replicates, for the figures and panels that the editor suggested. We also include a brief Matlab script for each of the supplementary data files that demonstrates how the data is plotted in the corresponding figure panels. The data is stored in .mat files, which is standard for our field and most compatible with our Matlab analysis/plotting code. We have uploaded the supplementary data files and code with the current submission as well as to our existing publicly available Zenodo repository (https://doi.org/10.5281/zenodo.11092491) that already contains all of the 3D printing files for the COMBO window. Although it is not explicitly requested, please note that all the raw data is available on Edmond Data Repository https://doi.org/10.17617/3.5KYYHK. We have updated the ***Data Availability*** section of the manuscript to reflect these additions to the Zenodo repository, as follows:

“All data needed to evaluate the conclusions in the paper are present in the paper or the Supplementary Materials. All relevant COMBO window files, supplementary data and plotting codes are freely available at the Zenodo repository https://doi.org/10.5281/zenodo.11092491. All raw fUS and two-photon data, along with the corresponding videographic recordings are freely available at the Edmond Data Repository https://doi.org/10.17617/3.5KYYHK. Additional data and software supporting the findings of this study are available from the corresponding authors upon reasonable request.”

**(C)** Please also ensure that each of the relevant figure legends in your manuscript include information on *WHERE THE UNDERLYING DATA CAN BE FOUND*, and ensure your supplemental data file/s has a legend.

**Response:** We have added a sentence to each main text and supplementary figure (where applicable) referencing in which supplementary data file the data for that figure can be found.

**(D)** Per journal policy, as the custom code that you have generated (page 24) to is important to support the conclusions of your manuscript, its deposition is required for acceptance. Please ensure that the code is sufficiently well documented and reusable, and that your Data Statement in the Editorial Manager submission system accurately describes where your code can be found.

Please note that we cannot accept sole deposition of code in GitHub, as this could be changed after publication. However, you can archive this version of your publicly available GitHub code to Zenodo. Once you do this, it will generate a DOI number, which you will need to provide in the Data Accessibility Statement (you are welcome to also provide the GitHub access information). See the process for doing this here: <https://docs.github.com/en/repositories/archiving-a-github-repository/referencing-and-citing-content>

**Response:** We have created custom Matlab scripts that generate plots for all of the supplementary data that we include with this submission. The same data and Matlab code can be found in our publicly available Zenodo repository (https://doi.org/10.5281/zenodo.11092491).

**(E)** Please ensure that your Data Statement in the submission system accurately describes where your data can be found and is in final format, as it will be published as written there.

**Response:** We confirm that our Data Statement in the submission system reflects the ***Data Availability*** section of the manuscript and accurately describes where all data can be found.
